# Genome-based surveillance reveals cross-transmission of MRSA ST59 between humans and retail livestock products in Hanzhong, China

**DOI:** 10.3389/fmicb.2024.1392134

**Published:** 2024-04-29

**Authors:** Wei Zhang, Xueshuo Wang, Linna Zhao, Yihai Gu, Yiwen Chen, Na Liu, Lin An, Li Bai, Yanjiong Chen, Shenghui Cui

**Affiliations:** ^1^Department of Immunology and Pathogenic Biology, College of Basic Medicine, Xi’an Jiaotong University Health Science Center, Xi’an, China; ^2^3201 Hospital, Hanzhong, China; ^3^National Institutes for Food and Drug Control, Beijing, China; ^4^China National Center for Food Safety Risk Assessment, Beijing, China

**Keywords:** interspecies transmission, livestock-associated methicillin-resistant *Staphylococcus aureus*, ST59, ST9, ST398

## Abstract

Methicillin-resistant *Staphylococcus aureus* (MRSA) has been recognized in hospitals, community and livestock animals and the epidemiology of MRSA is undergoing a major evolution among humans and animals in the last decade. This study investigated the prevalence of MRSA isolates from ground pork, retail whole chicken, and patient samples in Hanzhong, China. The further characterization was performed by antimicrobial susceptibility testing and in-depth genome-based analysis to identify the resistant determinants and their phylogenetic relationship. A total of 93 MRSA isolates were recovered from patients (*n* = 67) and retail livestock products (*n* = 26) in Hanzhong, China. 83.9% (78/93) MRSA isolates showed multiple drug resistant phenotype. Three dominant livestock-associated methicillin-resistant *Staphylococcus aureus* (LA-MRSA) sequence types were identified: ST59-t437 (*n* = 47), ST9-t899 (*n* = 10) and ST398 (*n* = 7). There was a wide variation among sequence types in the distribution of tetracycline-resistance, *scn*-negative livestock markers and virulence genes. A previous major human MRSA ST59 became the predominant interspecies MRSA sequence type among humans and retail livestock products. A few LA-MRSA isolates from patients and livestock products showed close genetic similarity. The spreading of MRSA ST59 among livestock products deserving special attention and active surveillance should be enacted for the further epidemic spread of MRSA ST59 in China. Data generated from this study will contribute to formulation of new strategies for combating spread of MRSA.

## 1 Introduction

Methicillin-resistant *Staphylococcus aureus* (MRSA) is one bacteria type that may cause numerous clinical manifestations ranging from mild skin and soft tissue infections to life-threatening fulminant invasive diseases (Turner et al., 2019; Tuffs et al., 2022). MRSA has been recognized in hospitals, community and livestock animals and the epidemiology of MRSA is undergoing a major evolution among humans and animals in the last decade (Chen et al., 2021; Yu et al., 2021). Since the livestock-associated MRSA (LA-MRSA) was first recognized in Europe in 2003, LA-MRSA has been identified in numerous countries around the world, including China (Cui et al., 2009; Silva et al., 2023). Multilocus sequence typing (MLST) analyzes seven constitutively expressed (housekeeping) genes that are essential to cellular functioning of organisms. LA-MRSA of sequence type (ST) 398 dominates in Europe, Australia and the United States, while LA-MRSA-ST9 is the main epidemic lineage in Asia (Yu et al., 2021; Silva et al., 2023). Several studies have identified specific LA-MRSA within the community acquired-MRSA category (Bisdorff et al., 2012; Mascaro et al., 2018; Sun et al., 2019). Recently, LA-MRSA-ST398 and other sequence types previously widely disseminated among human beings (such as ST59) were also identified in livestock animals and meat samples from China (Wang et al., 2014; Li et al., 2021; Zhang et al., 2021).

Because of the huge volume of livestock animals and the consumption of livestock products in the community, there is a concern that MRSA may be spreading and concentrating in livestock animals with subsequent dissemination into the community population through contact with livestock, farm environment or retail livestock products (Yu et al., 2021; Li et al., 2022). Especially retail livestock products, such as pork and poultry, are susceptible to MRSA contamination during slaughtering process and can become an ideal media of MRSA transmission to the kitchens (Hennekinne et al., 2012; Li et al., 2021).

In 2009, LA-MRSA-ST9 was firstly recognized from the swine and farm worker samples in Hanzhong city, Shaanxi province and all MRSA isolates in the previous study were grouped into ST9-spa899 (Cui et al., 2009). Hanzhong city lies in the center of the Hanzhong Basin. The south of the city is the Daba mountains and the north of the city is the Qinling mountains which limits the human migration and economic exchange with other areas. The location factor makes this city an ideal place for LA-MRSA transmission study. Since the pilot study in 2009 (Cui et al., 2009), no follow-up study was conducted on the transmission of LA-MRSA in this area.

The objective of this study was to determine the prevalence of MRSA isolates in ground pork, retail whole chicken and patient samples in Hanzhong city. The isolates were further characterized by in-depth genome-based analysis to identify the resistant determinants and their phylogenetic relationship.

## 2 Materials and methods

### 2.1 Sample collection and MRSA isolation

From July 2019 to May 2020, retail ground pork (*n* = 88) and retail whole chicken carcasses (*n* = 87) were collected from seven supermarkets in Hanzhong, China. Each supermarket was visited once a month. On each sampling day, no more than two whole chicken carcasses or two ground pork samples were randomly selected from each sampling site. All samples were transported to the laboratory and processed within 4 h. Each whole chicken carcass was immediately aseptically removed from the package and placed in a 3500 stomach bag (Seward, UK) followed by the addition of 500-mL buffered peptone water (BPW; Becton-Dickinson, Beijing, China). The bag was manually massaged for 3–5 min and the rinse were used for MRSA isolation. 25 ml of the rinse or 25 g of ground pork samples were added into 225 ml enrichment broth containing 1% tryptone, 7.5% sodium chloride, 1% mannitol and 0.25% yeast extract and incubated at 35 ± 1°C. After 22–24 h incubation, a loopful of the culture was inoculated onto selective MRSA agar plates (BBL CHROMagar MRSA) and incubated at 35 ± 1°C for 24–48 h. Purple colonies on the selective plates were screened for coagulase activity. All MRSA isolates were confirmed by the API Staph ID test (BioMe′rieux, Beijing, China) and PCR screening for the carriage of *nuc* and *mecA* (Merlino et al., 2002). One confirmed MRSA isolate from each sample was selected for further study. All MRSA isolates were kept in brain heart infusion broth (BD, China) with 50% glycerol at −80°C freezer for further analysis.

### 2.2 Collection of MRSA isolates from patients

MRSA isolates from patients were collected from the 3201 hospital which is the largest hospital in Hanzhong, Shaanxi, China. During the food sample collection, this hospital tested 7033 independent blood samples, 3517 cerebrospinal fluid samples, 128 wound secretion samples from inpatients for bacteria infection and 406 fecal samples from outpatients for *S. aureus*. The MRSA isolates from outpatient and inpatient samples were obtained and included in this study.

### 2.3 Antibiotic susceptibility testing

The antimicrobial susceptibility of all the MRSA isolates was determined by the micro-broth dilution method and interpreted according to the clinical and laboratory standards institute guidelines (Clinical and Laboratory Standards Institute [CLSI], 2022). The MICs of 13 antimicrobials were measured, including oxacillin (OXA), gentamicin (GEN), erythromycin (ERY), clindamycin (CLI), levofloxacin (LEV), vancomycin (VAN), teicoplanin (TEC), linezolid (LZD), trimethoprim-sulfamethoxazole (SXT), rifampin (RIF), nitrofurantoin (NIT), daptomycin (DAP) and tetracycline (TET). *S. aureus* ATCC 29213 was included as the quality control organism in antimicrobial susceptibility test to ensure that the concentration for each antimicrobial agent was properly controlled.

### 2.4 Genome sequencing and assembly

Bacterial genomic DNA of each isolate was extracted from 2 mL fresh culture using QIAamp DNA Mini Kit (Qiagen, Germany) following manufacturer’s instructions. DNA was subjected to quality control by visualizing electrophoresis products on a 1% agarose gel and quantifying them using a Qubit fluorometer (Invitrogen, Shanghai, China). WGS was performed with massively parallel sequencing (MPS) Illumina technology at Beijing Novogene Bioinformatics Technology Co., Ltd. A paired-end library with a 350 bp insert size was constructed and sequenced by Illumina NovaSeq using PE150 strategy. Illumina PCR adapter reads and low-quality reads were filtered by Readfq (version:10) and the filtered reads were assembled using SOAP denovo (version 2.04), SPAdes (version 3.10.0) and Abyss (version 1.3.7) to generate scaffolds (Li et al., 2008; Simpson et al., 2009; Bankevich et al., 2012) which were integrated by CISA software (Lin and Liao, 2013). The initial assembly results were optimized and matched using Gapclose (version 1.12) software to obtain the final assembly results (Luo et al., 2012). WGS data of 93 MRSA isolates were deposited into GenBank under BioProject accession number PRJNA966921 (Supplementary Table 3).

### 2.5 Bioinformatic analysis

The assembled contigs were subjected and analyzed on publicly available ResFinder 4.1, VirulenceFinder 2.0, MLST 2.0, spaTyper 1.0 and SCCmecFinder 1.2 server using default thresholds from the Center for Genomic Epidemiology (CGE) (Camacho et al., 2009; Larsen et al., 2012; Bartels et al., 2014; Bortolaia et al., 2020). The core genome alignment and SNPs calculation of genome in this research was performed using the snippy pipeline (version 4.4.5).^1^ The maximum likelihood tree was generated using the iqtree pipeline (version 2.1.2) (Kalyaanamoorthy et al., 2017) by including online isolates (Supplementary Table 4) and visualized using iTOL (Letunic and Bork, 2016).

### 2.6 Statistical analysis

The chi-square test was used to determine differences in the resistance rate of *S. aureus*. All statistical analyses were performed using the SPSS 18.0 software package.

## 3 Results

### 3.1 MRSA isolates isolation and confirmation

26 MRSA isolates were recovered from retailed ground pork (13/88) and retail whole chicken carcasses (13/87). A total of 67 MRSA isolates from outpatient fecal swabs (*n* = 18) and inpatient blood (*n* = 28), cerebrospinal fluid (*n* = 1) and wound secretion (*n* = 20) samples were obtained. All 93 isolates harbored *nuc* and *mecA*.

### 3.2 Susceptibility of MRSA isolates

All 93 MRSA isolates were resistant to oxacillin and susceptible to daptomycin, linezolid, nitrofurantoin, teicoplanin and vancomycin. Most MRSA isolates were also resistant to erythromycin (86.0%, 80/93), clindamycin (75.3%, 70/93) and tetracycline (50.5%, 47/93). 76.9% (20/26) food isolates were resistant to tetracycline which was significantly higher than the human isolates (40.3%, 27/67) (*P* < 0.01) and similar trend was found for clindamycin (Table 1). 83.9% (78/93) MRSA isolates showed multiple drug resistant phenotype. CLI-ERY-OXA (*n* = 30) and CLI-ERY-OXA-TET (*n* = 22) were two predominant multidrug resistant phenotypes. Only two of the CLI-ERY-OXA multidrug resistant isolates were from retail livestock product samples, whereas ten of the CLI-ERY-OXA-TET multidrug resistant isolates were from retail livestock product samples and the difference of this distribution pattern was significant (*P* < 0.05) (Supplementary Table 2).

**TABLE 1:** Resistance phenotypes of MRSA isolates (*n* = 93) from food and patient samples, Hanzhong, China.

Antimicrobial agents	MIC* mg/L	No. (%) of resistant isolates					
		Total (*N* = 93)	Patients (*N* = 67)	Food(*N* = 26)	ST59(*N* = 57)	ST9(*N* = 10)	ST398(*N* = 7)
Clindamycin	≥4	70 (75.3)	47 (70.1)	23 (88.5)	49 (86.0)	10 (100)	4 (57.1)
Erythromycin	≥8	80 (86.0)	56 (83.6)	24 (92.3)	54 (94.7)	10 (100)	4 (57.1)
Gentamicin	≥16	21 (22.6)	12 (18.0)	9 (34.6)	1 (1.8)	10 (100)	0 (0.0)
Levofloxacin	≥4	16 (17.2)	10 (15.0)	6 (23.1)	2 (3.5)	5 (50.0)	0 (0.0)
Tetracycline	≥16	47 (50.5)	27 (40.3)	20 (76.9)	24 (42.1)	10 (100)	3 (42.9)
Rifampin	≥4	10 (10.8)	10 (14.9)	0 (0.0)	1 (1.8)	0 (0.0)	0 (0.0)
Trimethoprim-sulfamethoxazole	≥4/76	10 (10.8)	1 (1.5)	9 (34.6)	0 (0.0)	9 (90.0)	0 (0.0)

*MIC, minimal inhibitory concentration.

### 3.3 MLST analysis of MRSA isolates

93 MRSA isolates were grouped into 11 STs, including ST59 (*n* = 57), ST9 (*n* = 10), ST239 (*n* = 9), ST398 (*n* = 7), ST88 (*n* = 3), ST6576 (*n* = 2), ST338 (*n* = 1), ST45 (*n* = 1), ST5 (*n* = 1), ST5052 (*n* = 1) and ST509 (*n* = 1). ST59 was the dominant sequence type among ground pork (*n* = 7/13), whole chicken (*n* = 6/13), outpatient fecal swabs (*n* = 15/18), inpatient sterile sites (*n* = 15/29) and wound secretion (*n* = 14/20) samples. ST9 (*n* = 10), ST398 (*n* = 7) and ST88 (*n* = 3) isolates were also identified from both food and patient samples (Supplementary Table 1).

### 3.4 Characterization and phylogenetic analysis of ST59 isolates

Eight resistance phenotypes were identified among 57 ST59 isolates. Among isolates of CLI-ERY-OXA resistant phenotype, 92.8% (26/28) isolates were sourced from human samples. However, among isolates of CLI-ERY-OXA-TET resistant phenotype, 57.9% (11/19) isolates were sourced from human samples and the distribution difference was highly significant (*P* < 0.01). One isolate from patient secretion sample showed CLI-ERY-GEN-LEV-OXA-RIF-TET resistant phenotype. *Staphylococcus* chromosomal cassette mec (*SCCmec*) *_type_IVa(2B)* was identified among 54 ST59 isolates. *SCCmec_type_Vb(5C2&5)* was identified in two isolates from inpatient blood samples and *SCCmec_type_IVg(2B)* was identified in one isolate from chicken sample. The common resistant determinants identified among ST59 isolates including *aph(3′)-III* (42/57), *blaZ* (49/57), *ermB* (46/57), *ermC* (13/57) and *tet(K)* (28/57) (Supplementary Table 1).

Six Staphylococcal protein a (*spa*) typing were identified among 57 ST59 MRSA isolates, including t437 (*n* = 47), t441 (*n* = 6), t13774 (*n* = 1), t3515 (*n* = 1), t4193 (*n* = 1) and t8391 (*n* = 1). *Spa* t437 was identified among ground pork (*n* = 7/7), whole chicken (*n* = 2/6), outpatient fecal swabs (*n* = 13/15), inpatient sterile sites (*n* = 14/15) and wound secretion (*n* = 11/14) samples. *Spa* t441 was identified among whole chicken (*n* = 1), outpatient fecal swabs (*n* = 2), inpatient wound secretion (*n* = 2) and sterile site (*n* = 1) samples.

**FIGURE 1:**
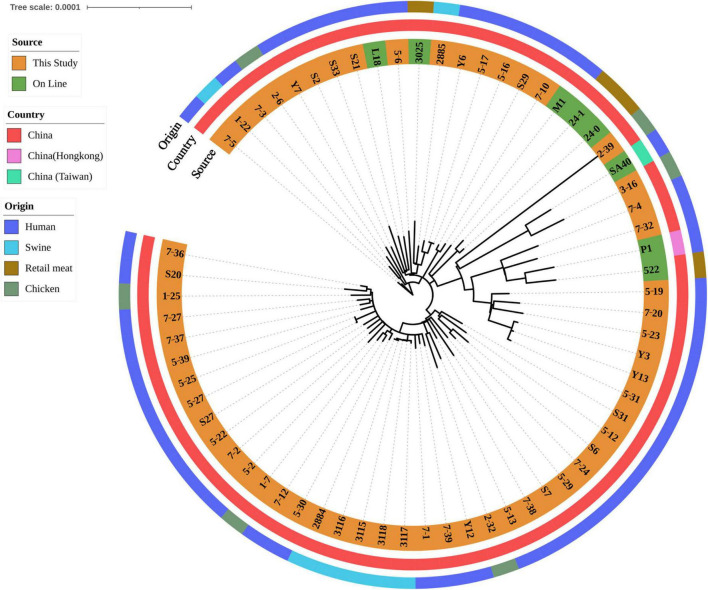
Phylogenetic relationship of ST59 isolates from retail livestock products and patients, Hanzhong, China. The genome sequences of 65 ST59 isolates were aligned through SNP analysis, including 8 online isolates. The information of online isolates was provided in Supplementary Table 4.

After SNP analysis, eight isolates harbored *lukF/S-PV* were grouped into one cluster, comprising isolates 5-23, 5-19, 7-20, Y3, 5-31, Y13, 7-4 and 7-32 (Figure 1). Human isolates from blood, fecal and wound secretion samples were crossly distributed. A cluster of five ground pork isolates (isolates 2884, 3115, 3116, 3117 and 3118) of CLI-ERY-OXA-TET multidrug resistant phenotype was identified. Two ground pork isolates were scattered among patient isolates with isolate 1-22 from ground pork showing a close relationship with isolate 7-5 from patient blood sample, while another ground pork isolate 2885 showed a close relationship with pork isolate 3025 of online reference. Six isolates from whole chicken carcasses were scattered among human isolates. Genetic similarities were found among five pairs of chicken and human isolates (Figure 1).

### 3.5 Characterization and phylogenetic analysis of ST9 isolates

ST9 isolates (*n* = 10) were identified from whole chicken (*n* = 6), ground pork (*n* = 3) and inpatient secretion (*n* = 1) samples. All ST9 isolates were resistant to clindamycin, erythromycin, gentamicin, oxacillin, tetracycline. Additionally, the strains were also intermediate or resistant to levofloxacin (MIC was found to be 2 or 4 μg/ml). Nine food isolates were also found to be resistant to trimethoprim/sulfamethoxazole. All 10 ST9 MRSA isolates displayed the same *spa* type (allelic profile, 07-16-23-02-34, t899) and contained *SCCmec_type_XII(9C2)*.

**FIGURE 2:**
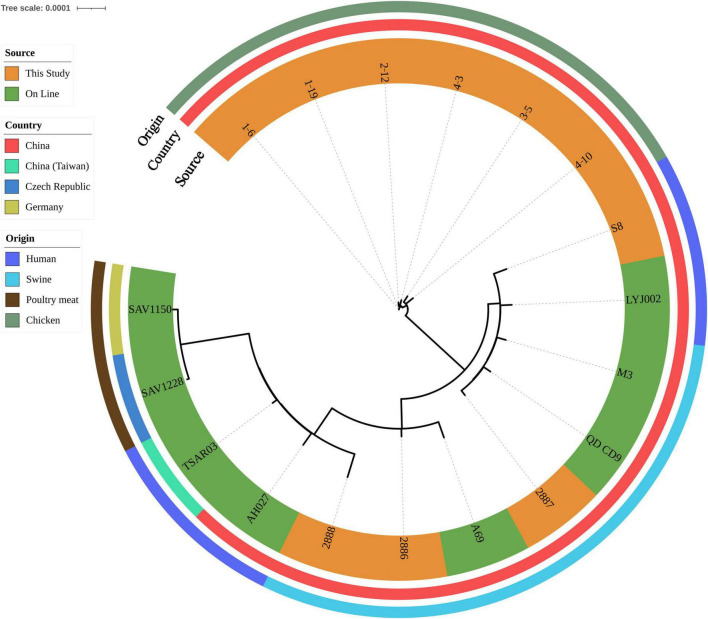
Phylogenetic relationship of ST9 isolates from retail livestock products and patients, Hanzhong, China. The genome sequences of 18 ST9 isolates were aligned through SNP analysis, including 8 online isolates. The information of online isolates was provided in Supplementary Table 4.

All isolates harbored the following resistant determinants: *aac(6′)*, *aadD*, *aph(2″)*, *blaZ*, *dfrG*, *erm(C)*, *lsa(E)*, *lnu(B)*, *mecA* and *tet(L)*. Eight isolates also harbored florfenicol-chloramphenicol resistant determinant *fexA* (Supplementary Table 1). After SNP analysis, six chicken isolates were grouped together. Genetic similarities were found between one retail ground pork isolate 2887 and one wound secretion isolate S8 (Figure 2).

### 3.6 Characterization and phylogenetic analysis of ST398 isolates

**FIGURE 3:**
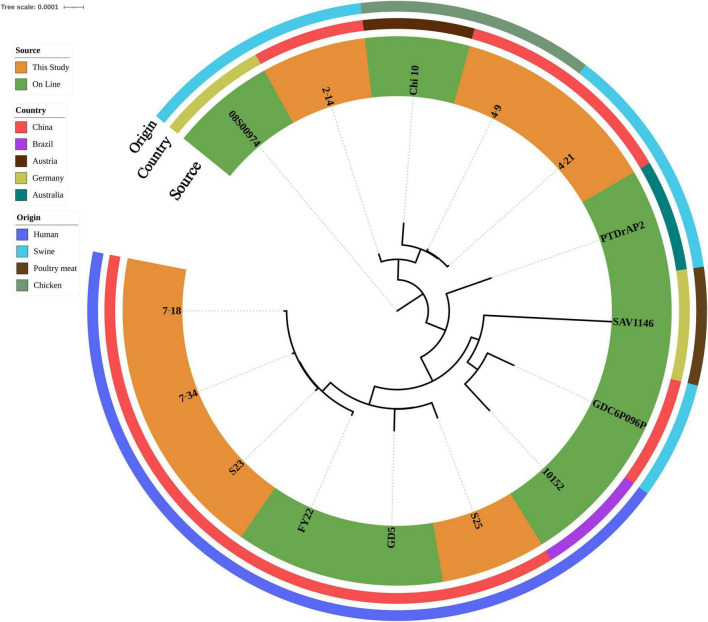
Phylogenetic relationship of ST398 isolates from retail livestock products and patients, Hanzhong, China. The genome sequences of 15 ST398 isolates were aligned through SNP analysis, including 8 online isolates. The information of online isolates was provided in Supplementary Table 4.

ST398 isolates (*n* = 7) were identified from ground pork (*n* = 2), whole chicken (*n* = 1), inpatient secretion (*n* = 2) and blood (*n* = 2) samples. All ST398 isolates (*n* = 7) were susceptible to gentamycin, linezolid, nitrofurantoin, rifampin, teicoplanin, tigecycline, trimethoprim/sulfamethoxazole, vancomycin and resistant to oxacillin. Clindamycin and tetracycline resistance were observed in three food isolates and two isolates were also resistant to erythromycin. All four human isolates were susceptible to tetracycline and two isolates were resistant to erythromycin and one isolate was also resistant to clindamycin. Four human isolates were grouped into three *spa* types: t034 (*n* = 2), t571(*n* = 1) and t1928 (*n* = 1). t034 (*n* = 2) and t1928 isolates contained *SCCmec_type_V(5C2)*, t571(*n* = 1) isolate contained *SCCmec_type_III(3A)*. All three food isolates shared the same *spa* type (t011) and contained *SCCmec_type_Vc(5C2&5)*. All ST398 isolates harbored *blaZ* and *mecA*. All three food isolates also harbored *dfrG*, *lnu(B)*, *lsa(E)*, *tet(K)* and *tet(M)* (Supplementary Table 1). After SNP analysis, four human isolates showed genetic similarities and were grouped independently from three food isolates (Figure 3).

### 3.7 Virulence genes among ST59, ST9 and ST398 isolates

All ST59, ST9, and ST398 isolates were found to contain the following virulence factors, including the metalloprotease (*aur*) and the hemolysins *hlgA*, *hlgB*, and *hlgC*. PVL was present in eight ST59-*spa* t437 human isolates (8/44, 18.2%) and six isolates were staphylokinase (*sak*) negative, but all the PVL negative ST59 MRSA isolates were *sak* positive. The distribution of *scn*, *sak* and enterotoxin encoding genes showed different patterns across different STs. All ST59 (*n* = 57) and patient ST398 (*n* = 4) isolates were *scn* positive, all ST9 (*n* = 10) and the food ST398 (*n* = 3) isolates were *scn* and *sak* negative.

Among ST59 isolates, *sak* was identified among 51 isolates and enterotoxins *seb*, *sek* and *seq* were identified among 50 isolates. Both *sak and* enterotoxins *seb*, *sek* and *seq* were identified among 45 isolates from ground pork (*n* = 7/7), whole chicken (*n* = 5/6), fecal swabs (*n* = 13/15), sterile sites (*n* = 10/15) and wound secretion (*n* = 10/14) samples. All ST9 isolates harbored enterotoxin gene *seo*, and eight ST9 food isolates also harbored more enterotoxin gene (*seg*, *sei*, *sem*, *sen* and *seu*), but these genes were absent in ST398 isolates (Supplementary Table 1).

### 3.8 Characterization of the remaining MRSA isolates

Except one isolate recovered from the ground pork sample, the other leftover MRSA isolates (*n* = 18) were recovered from patient samples. These 19 isolates were grouped into eight STs. All ST239 (*n* = 9) isolates showed multiple resistant phenotypes and carried *SCCmec_type_III(3A)* and were grouped into *spa* t030 (*n* = 5), *spa* t459 (*n* = 4) types. *SCCmec_type_IV* (*n* = 8) and *SCCmec_type_V* (*n* = 3) were identified among the other isolates. Except one ST45 isolate from the fecal sample, the other MRSA isolates (*n* = 18) were all *scn* and *sak* positive. *lukE/D* was present in 15 isolates.

## 4 Discussion

From patients and retail livestock products in Hanzhong, China, three dominant LA-MRSA sequence types were identified: ST59-t437 (*n* = 47), ST9-t899 (*n* = 10), and ST398 (*n* = 7). There was a wide variation among sequence types in the distribution of tetracycline-resistance, *scn*-negative livestock markers and virulence genes, indicating different origins and evolutionary processes. We found that in Hanzhong, China, a major human MRSA ST59, not LA-MRSA ST9 or ST398, became the predominant interspecies MRSA sequence type among humans and retail livestock products. Some MRSA-ST59 isolates from patients and livestock products showed close genetic similarity. Our study further indicated that certain human MRSA may become LA-MRSA and transfer from humans to livestock animals or vice versa, which demonstrated the importance of continuous MRSA surveillance among humans and livestock animals.

Our data showed ST59 MRSA was dominant not only among patients in Hanzhong (44/67), but also among livestock product isolates (13/26). Initially being reported in North America in the early 2000s (Enright et al., 2002), ST59 MRSA has gradually been replacing ST239 and ST5 human clones and become the predominant sequence type in most hospitals of China since 2010 (Li et al., 2018; Jin et al., 2021; Wang et al., 2022). However, ST59 MRSA remains geographically confined and is low prevalence in Europe and North America (Pimentel de Araujo et al., 2021; Di Gregorio et al., 2023). Previous studies have shown ST9 is the dominant LA-MRSA in China and other Asian countries as opposed to ST398 in Europe and North America (Silva et al., 2023). Recent studies have also reported ST59 MRSA isolates were scattered among livestock animals in China (Wang et al., 2021; Li et al., 2022), our data indicated that the detection rate of ST59 in livestock products in Hanzhong was higher than ST9 and 398 isolates. ST59 MRSA isolates displayed a greater overall phylogenetic diversity than LA-MRSA ST9 isolates did which indicated the multiple origins of ST59 isolates or higher adaptability within different hosts, suggesting that the ST59 isolates might undergo multiple and continuous evolutionary events. Usually, ST59 isolates carry *SCCmec_type_IV* (55/57) or *SCCmec_type_V*, both are smaller *SCCmec* cassettes that may reduce the host fitness burden. Coculture experiment in a previous study showed that ST59 isolates displayed higher growth rates and competitive capacity than MRSA ST239 *in vitro* which provided further evidence that ST59 clones may have a better capacity for surviving outside the host and promote ST59 transmission among livestock animals and human beings (Li et al., 2018). In this study, some ST59 isolates from livestock products were scattered among patient isolates in phylogenetic tree suggesting that frequent exchanges might occur between livestock animals and patients. Because of the higher adaptability and competitive power of ST59 isolates, a detailed study of the genetic basis for the successful dissemination of ST59 among both humans and livestock animals in China should be conducted.

Multiple livestock-association markers, such as *scn-*negative, tetracycline-resistance, CC9, CC398 have been reported in different studies (Verkaik et al., 2011; Cuny et al., 2015). There is growing evidence that accessory genes carried by prophages of *S. aureus* significantly modulate bacterial fitness as they carry multiple virulence factors (VFs). These VFs include human immune evasion cluster (IEC) comprising the genes sak, chp, scn and sea/sep (Nepal et al., 2021). As a marker of IEC, *scn* has been recognized as the indicator of human *S. aureus* isolates and might be useful for differentiating livestock isolates from human isolates (Rinsky et al., 2013). In this study, all human ST398 (*n* = 4) isolates were *scn* positive, but all ST9 (*n* = 10) and food ST398 (*n* = 3) isolates were *scn* negative. This was consistent with a recent study that the loss of IEC might happen after their shift from human to animals because antimicrobials in feed can induce *scn* prophage loss (Allen et al., 2011; Price et al., 2012; Yu et al., 2021). All food (*n* = 13) ST59 isolates in this study were *scn-*positive which indicated these isolates might jump from human into the livestock animals recently. A recent study also found *scn-*positive ST59 was the major MRSA among Yak (Bos grunniens) herds in Tibetan, China (Zou et al., 2022). A continuous surveillance study should be carried out to find out how long ST59 isolates would keep *scn* after it was transmitted to livestock hosts and the factors that might influence the speed of *scn* loss.

Tetracycline-resistance is another livestock-association marker different from human isolates (Rinsky et al., 2013). Tetracyclines are a class of broad-spectrum antibiotics, including naturally occurring and semi-synthetic tetracyclines (Roberts, 2003). Due to their side-effects, the use of tetracyclines is limited in human clinics, but they are still been used globally as important infection treatment measures and growth promoters among livestock animals which promoted the tetracycline resistant determinants transmission in livestock products (Inglis et al., 2019; Chen et al., 2023). Since animals cannot fully absorb or metabolize tetracyclines, they excrete a significant fraction of such drugs or of their breakdown products into the environment via feces or urine which may also promote the transmission of resistant determinants (Mackie et al., 2006). More than 30 tetracycline specific resistant determinants have been recognized and some of them can be transferred horizontally among different bacteria through mobile genetic elements, such as plasmids, phages or integrons (Roberts, 2003). In this study, the food isolates (20/26) showed significantly higher frequency of tetracycline resistance than isolates from human beings (27/67) which indicated the food isolates circulating among livestock animals for considerable time. The genotypes of tetracycline resistant determinants were corresponding to the sequence types which further indicated different evolution processes of these isolates.

Genetic advantages might contribute to the replacement of LA-MRSA from ST9 to ST59 locally. Diversified genetic context was found among 57 ST59 MRSA isolates which were group into six *spa* types with t437 (*n* = 47) as the local dominant LA-MRSA type which was more diversified than ST9 (*n* = 10) isolates that were all belonged to *spa* t899 (Cui et al., 2009; Wang et al., 2017). Other studies also reported much more diverse genetic context of ST59 isolates that probably reflected active and extensive genetic recombination of this clone (Jin et al., 2021; Wang et al., 2021). The diversified genetic context might have more exchange and adaption opportunities and speed up the transmission of this clone. More virulence factors might be another advantage that could contribute to ST59 higher transmission compacity than ST9 isolates (Li et al., 2016). ST9 was originated from human-adapted strains which had lost genes related to the evasion of the immune system (Yu et al., 2021). In this study, all ST9 (*n* = 10) isolates were *scn* and *sak* negative which further confirmed this theory.

There are several shortages of this study. All MRSA isolates were from patients and retail livestock product samples in Hanzhong city, China and no isolates recovered from livestock animals were included. Limited isolates of ST9 and ST398 were analyzed from human and retail livestock product samples in this study. More isolates from other areas should be analyzed to confirm the distribution patterns found in this study.

## 5 Conclusion

Because of the high adaption and transmission capacity, MRSA ST59 may be widespread and become the dominant clones among livestock animals in the future. Therefore, active surveillance should be enacted for the further epidemic spread of MRSA ST59 in China.

## Data availability statement

The names of the repository/repositories and accession number(s) can be found below: https://www.ncbi.nlm.nih.gov/, PRJNA966921.

## Ethics statement

The studies involving humans were approved by 3201 Hospital Ethics Committee, Affiliated with 3201 Hospital. The studies were conducted in accordance with the local legislation and institutional requirements. Written informed consent for participation was not required from the participants or the participants’ legal guardians/next of kin in accordance with the national legislation and institutional requirements.

## Author contributions

WZ: Conceptualization, Methodology, Software, Validation, Visualization, Formal analysis, Investigation, Resources, Data curation, Funding acquisition, Writing – original draft, Writing – review and editing. XW: Formal analysis, Visualization, Writing – original draft. LZ: Formal analysis, Visualization, Writing – original draft. YG: Methodology, Resources, Writing – original draft. YiC: Formal analysis, Writing – original draft. NL: Formal analysis, Writing – original draft. LA: Formal analysis, Writing – original draft. LB: Conceptualization, Methodology, Writing – review and editing. YaC: Conceptualization, Data curation, Investigation, Methodology, Project administration, Resources, Software, Writing – review and editing, Supervision, Validation. SC: Conceptualization, Methodology, Software, Writing – review and editing, Supervision, Validation, Funding acquisition, Project administration.

## Footnotes

1
https://github.com/tseemann/snippy

## References

[B1] Allen H. K. Looft T. Bayles D. O. Humphrey S. Levine U. Y. Alt D. (2011). Antibiotics infeed induce prophages in swine fecal microbiomes. *mBio* 2:e00260-11. 10.1128/mbio.00260-11 22128350 PMC3225969

[B2] Bankevich A. Nurk S. Antipov D. Gurevich A. A. Dvorkin M. Kulikov A. S. (2012). SPAdes: A new genome assembly algorithm and its applications to single-cell sequencing. *J. Comput. Biol.* 19 455–477. 10.1089/cmb.2012.0021 22506599 PMC3342519

[B3] Bartels M. D. Petersen A. Worning P. Nielsen J. B. Larner-Svensson H. Johansen H. K. (2014). Comparing whole-genome sequencing with Sanger sequencing for spa typing of methicillin-resistant *Staphylococcus aureus*. *J. Clin. Microbiol.* 52 4305–4308. 10.1128/jcm.01979-14 25297335 PMC4313303

[B4] Bisdorff B. Scholholter J. L. Claussen K. Pulz M. Nowak D. Radon K. (2012). MRSA-ST398 in livestock farmers and neighbouring residents in a rural area in Germany. *Epidemiol. Infect.* 140 1800–1808. 10.1017/S0950268811002378 22313681

[B5] Bortolaia V. Kaas R. S. Ruppe E. Roberts M. C. Schwarz S. Cattoir V. (2020). ResFinder 4.0 for predictions of phenotypes from genotypes. *J. Antimicrob. Chemother.* 75 3491–3500. 10.1093/jac/dkaa345 32780112 PMC7662176

[B6] Camacho C. Coulouris G. Avagyan V. Ma N. Papadopoulos J. Bealer K. (2009). BLAST+: Architecture and applications. *BMC Bioinformatics* 10:421. 10.1186/1471-2105-10-421 20003500 PMC2803857

[B7] Chen H. Yin Y. van Dorp L. Shaw L. P. Gao H. Acman M. (2021). Drivers of methicillin-resistant *Staphylococcus aureus* (MRSA) lineage replacement in China. *Genome Med.* 13:171. 10.1186/s13073-021-00992-x 34711267 PMC8555231

[B8] Chen Y. Sun L. Hong Y. Chen M. Zhang H. Peng Y. (2023). Exploring the third-generation tetracycline resistance of multidrug-resistant livestock-associated methicillin-resistant *Staphylococcus aureus* ST9 across healthcare settings in China. *J. Antimicrob. Chemother.* 78 1871–1881. 10.1093/jac/dkad174 37287125 PMC10393890

[B9] Clinical and Laboratory Standards Institute [CLSI] (2022). *Performance standards for antimicrobial susceptibility testing. CLSI approved standard M100-S32.* Wayne, IL: Clinical and Laboratory Standards Institute.

[B10] Cui S. Li J. Hu C. Jin S. Li F. Guo Y. (2009). Isolation and characterization of methicillin-resistant *Staphylococcus aureus* from swine and workers in China. *J. Antimicrob. Chemother.* 64 680–683. 10.1093/jac/dkp275 19684078

[B11] Cuny C. Abdelbary M. Layer F. Werner G. Witte W. (2015). Prevalence of the immune evasion gene cluster in *Staphylococcus aureus* CC398. *Vet. Microbiol.* 177 219–223. 10.1016/j.vetmic.2015.02.031 25778546

[B12] Di Gregorio S. Vielma J. Haim M. S. Rago L. Campos J. Kekre M. (2023). Genomic epidemiology of *Staphylococcus aureus* isolated from bloodstream infections in South America during 2019 supports regional surveillance. *Microb. Genom.* 9:mgen001020. 10.1099/mgen.0.001020 37227244 PMC10272885

[B13] Enright M. C. Robinson D. A. Randle G. Feil E. J. Grundmann H. Spratt B. G. (2002). The evolutionary history of methicillin-resistant *Staphylococcus aureus* (MRSA). *Proc. Natl. Acad. Sci. U.S.A.* 99 7687–7692. 10.1073/pnas.122108599 12032344 PMC124322

[B14] Hennekinne J. A. De Buyser M. L. Dragacci S. (2012). *Staphylococcus aureus* and its food poisoning toxins: Characterization and outbreak investigation. *FEMS Microbiol. Rev.* 36 815–836. 10.1111/j.1574-6976.2011.00311.x 22091892

[B15] Inglis G. D. Gusse J. F. House K. E. Shelton T. G. Taboada E. N. (2019). Tetracycline resistant *Campylobacter jejuni* subtypes emanating from beef cattle administered non-therapeutic chlortetracycline are longitudinally transmitted within the production continuum but are not detected in ground beef. *Microorganisms* 8:23. 10.3390/microorganisms8010023 31877744 PMC7022225

[B16] Jin Y. Zhou W. Zhan Q. Zheng B. Chen Y. Luo Q. (2021). Genomic epidemiology and characterization of methicillin-resistant *Staphylococcus aureus* from bloodstream infections in China. *mSystems* 6:e0083721. 10.1128/mSystems.00837-21 34726482 PMC8562482

[B17] Kalyaanamoorthy S. Minh B. Q. Wong T. K. F. von Haeseler A. Jermiin L. S. (2017). ModelFinder: Fast model selection for accurate phylogenetic estimates. *Nat. Methods* 14 587–589. 10.1038/nmeth.4285 28481363 PMC5453245

[B18] Larsen M. V. Cosentino S. Rasmussen S. Friis C. Hasman H. Marvig R. L. (2012). Multilocus sequence typing of total-genome-sequenced bacteria. *J. Clin. Microbiol.* 50 1355–1361. 10.1128/jcm.06094-11 22238442 PMC3318499

[B19] Letunic I. Bork P. (2016). Interactive tree of life (iTOL) v3: An online tool for the display and annotation of phylogenetic and other trees. *Nucleic Acids Res.* 44 W242–W245. 10.1093/nar/gkw290 27095192 PMC4987883

[B20] Li H. Tang T. Stegger M. Dalsgaard A. Liu T. Leisner J. J. (2021). Characterization of antimicrobial-resistant *Staphylococcus aureus* from retail foods in Beijing, China. *Food Microbiol.* 93:103603. 10.1016/j.fm.2020.103603 32912578

[B21] Li M. Dai Y. Zhu Y. Fu C. L. Tan V. Y. Wang Y. (2016). Virulence determinants associated with the Asian community-associated methicillin-resistant *Staphylococcus aureus* lineage ST59. *Sci. Rep.* 6:27899. 10.1038/srep27899 27296890 PMC4906288

[B22] Li R. Li Y. Kristiansen K. Wang J. (2008). SOAP: Short oligonucleotide alignment program. *Bioinformatics* 24 713–714. 10.1093/bioinformatics/btn025 18227114

[B23] Li S. Sun S. Yang C. Chen H. Yin Y. Li H. (2018). The changing pattern of population structure of *Staphylococcus aureus* from bacteremia in China from 2013 to 2016: ST239-030-MRSA replaced by ST59-t437. *Front. Microbiol.* 9:332. 10.3389/fmicb.2018.00332 29535697 PMC5835333

[B24] Li Y. Li W. Pan Y. Liu C. Liang S. Zeng Z. (2022). The emergence and molecular study of methicillin-resistant *Staphylococcus aureus* ST239, ST59, ST9, and ST630 in food animals, Chongqing, China. *Vet. Microbiol.* 265:109329. 10.1016/j.vetmic.2021.109329 35030381

[B25] Lin S. H. Liao Y. C. (2013). CISA: Contig integrator for sequence assembly of bacterial genomes. *PLoS One* 8:e60843. 10.1371/journal.pone.0060843 23556006 PMC3610655

[B26] Luo R. Liu B. Xie Y. Li Z. Huang W. Yuan J. (2012). SOAPdenovo2: An empirically improved memory-efficient short-read de novo assembler. *Gigascience* 1:18. 10.1186/2047-217X-1-18 23587118 PMC3626529

[B27] Mackie R. I. Koike S. Krapac I. Chee-Sanford J. Maxwell S. Aminov R. I. (2006). Tetracycline residues and tetracycline resistance genes in groundwater impacted by swine production facilities. *Anim. Biotechnol.* 17 157–176. 10.1080/10495390600956953 17127527

[B28] Mascaro V. Leonetti M. Nobile C. G. A. Barbadoro P. Ponzio E. Recanatini C. (2018). Prevalence of livestock-associated methicillin-resistant *Staphylococcus aureus* (LA-MRSA) among farm and slaughterhouse workers in Italy. *J. Occup. Environ. Med.* 60 e416–e425. 10.1097/JOM.0000000000001385 29933320

[B29] Merlino J. Watson J. Rose B. Beard-Pegler M. Gottlieb T. Bradbury R. (2002). Detection and expression of methicillin/oxacillin resistance in multidrug-resistant and non-multidrug-resistant *Staphylococcus aureus* in Central Sydney, Australia. *J. Antimicrob. Chemother.* 49 793–801. 10.1093/jac/dkf021 12003973

[B30] Nepal R. Houtak G. Shaghayegh G. Bouras G. Shearwin K. Psaltis A. J. (2021). Prophages encoding human immune evasion cluster genes are enriched in *Staphylococcus aureus* isolated from chronic rhinosinusitis patients with nasal polyps. *Microb. Genom.* 7:000726. 10.1099/mgen.0.000726 34907894 PMC8767322

[B31] Pimentel de Araujo F. Monaco M. Del Grosso M. Pirolo M. Visca P. Pantosti A. (2021). *Staphylococcus aureus* clones causing osteomyelitis: A literature review (2000-2020). *J. Glob. Antimicrob. Resist.* 26 29–36. 10.1016/j.jgar.2021.03.030 33965630

[B32] Price L. B. Stegger M. Hasman H. Aziz M. Larsen J. Andersen P. S. (2012). *Staphylococcus aureus* CC398: Host adaptation and emergence of methicillin resistance in livestock. *mBio* 3 e305–e311. 10.1128/mbio.00305-11 22354957 PMC3280451

[B33] Rinsky J. L. Nadimpalli M. Wing S. Hall D. Baron D. Price L. B. (2013). Livestock-associated methicillin and multidrug resistant *Staphylococcus aureus* is present among industrial, not antibiotic-free livestock operation workers in North Carolina. *PLoS One* 8:e67641. 10.1371/journal.pone.0067641 23844044 PMC3699663

[B34] Roberts M. C. (2003). Tetracycline therapy: Update. *Clin. Infect. Dis.* 36 462–467. 10.1086/367622 12567304

[B35] Silva V. Araujo S. Monteiro A. Eira J. Pereira J. E. Maltez L. (2023). *Staphylococcus aureus* and MRSA in livestock: Antimicrobial resistance and genetic lineages. *Microorganisms* 11:124. 10.3390/microorganisms11010124 36677414 PMC9865216

[B36] Simpson J. T. Wong K. Jackman S. D. Schein J. E. Jones S. J. Birol I. (2009). ABySS: A parallel assembler for short read sequence data. *Genome Res.* 19 1117–1123. 10.1101/gr.089532.108 19251739 PMC2694472

[B37] Sun C. Chen B. Hulth A. Schwarz S. Ji X. Nilsson L. E. (2019). Genomic analysis of *Staphylococcus aureus* along a pork production chain and in the community, Shandong Province, China. *Int. J. Antimicrob. Agents* 54 8–15. 10.1016/j.ijantimicag.2019.03.022 30959181

[B38] Tuffs S. W. Goncheva M. I. Xu S. X. Craig H. C. Kasper K. J. Choi J. (2022). Superantigens promote *Staphylococcus aureus* bloodstream infection by eliciting pathogenic interferon-gamma production. *Proc. Natl. Acad. Sci. U.S.A.* 119:e2115987119. 10.1073/pnas.2115987119 35165181 PMC8872782

[B39] Turner N. A. Sharma-Kuinkel B. K. Maskarinec S. A. Eichenberger E. M. Shah P. P. Carugati M. (2019). Methicillin-resistant *Staphylococcus aureus*: An overview of basic and clinical research. *Nat. Rev. Microbiol.* 17 203–218. 10.1038/s41579-018-0147-4 30737488 PMC6939889

[B40] Verkaik N. J. Benard M. Boelens H. A. de Vogel C. P. Nouwen J. L. Verbrugh H. A. (2011). Immune evasion cluster-positive bacteriophages are highly prevalent among human *Staphylococcus aureus* strains, but they are not essential in the first stages of nasal colonization. *Clin. Microbiol. Infect.* 17 343–348. 10.1111/j.1469-0691.2010.03227.x 20370801

[B41] Wang B. Xu Y. Zhao H. Wang X. Rao L. Guo Y. (2022). Methicillin-resistant *Staphylococcus aureus* in China: A multicentre longitudinal study and whole-genome sequencing. *Emerg. Microbes Infect.* 11 532–542. 10.1080/22221751.2022.2032373 35060838 PMC8843102

[B42] Wang W. Baker M. Hu Y. Xu J. Yang D. Maciel-Guerra A. (2021). Whole-genome sequencing and machine learning analysis of *Staphylococcus aureus* from multiple heterogeneous sources in China reveals common genetic traits of antimicrobial resistance. *mSystems* 6:e0118520. 10.1128/msystems.01185-20 34100643 PMC8579812

[B43] Wang W. Liu F. Baloch Z. Zhang C. S. Ma K. Peng Z. X. (2017). Genotypic characterization of methicillin-resistant *Staphylococcus aureus* isolated from pigs and retail foods in China. *Biomed. Environ. Sci.* 30 570–580. 10.3967/bes2017.076 28807097

[B44] Wang X. Li G. Xia X. Yang B. Xi M. Meng J. (2014). Antimicrobial susceptibility and molecular typing of methicillin-resistant *Staphylococcus aureus* in retail foods in Shaanxi, China. *Foodborne Pathog. Dis.* 11 281–286. 10.1089/fpd.2013.1643 24404781

[B45] Yu F. Cienfuegos-Gallet A. V. Cunningham M. H. Jin Y. Wang B. Kreiswirth B. N. (2021). Molecular evolution and adaptation of livestock-associated methicillin-resistant *Staphylococcus aureus* (LA-MRSA) sequence type 9. *mSystems* 6:e0049221. 10.1128/msystems.00492-21 34156294 PMC8269235

[B46] Zhang T. Jia M. Cheng Y. Zhang W. Lu Q. Guo Y. (2021). First report of ST9-MRSA-XII from a chicken farm in China. *J. Glob. Antimicrob. Resist.* 27 292–293. 10.1016/j.jgar.2021.10.018 34788688

[B47] Zou G. Matuszewska M. Bai F. Wang S. Wang S. Li H. (2022). Genomic analyses of *Staphylococcus aureus* isolated from yaks in Ganzi Tibetan autonomous prefecture, China. *J. Antimicrob. Chemother.* 77 910–920. 10.1093/jac/dkac011 35099017

