# Supplementary material for: Genome-based surveillance reveals cross-transmission of MRSA ST59 between humans and retail livestock products in Hanzhong, China
Source: Front Microbiol. 2024 Apr 29;15:1392134. doi: 10.3389/fmicb.2024.1392134 (PMC11089119; doi:10.3389/fmicb.2024.1392134)
Supplement: Supplementary file 1 [file Table_1.docx]

**Supplementary Table 1 Molecular characteristics of food and patient MRSA isolates from Hanzhong, China**

|  | Strains | Origin | MLST | *Spa* types | *SCCmec* | Resistant genes | Virulence genes | Genebank Accession |
| --- | --- | --- | --- | --- | --- | --- | --- | --- |
| 1 | 7-1 | Blood | ST59 | t437 | IVa(2B) | *aph(3')-III-blaZ-erm(B)-mecA-tet(K)* | *aur-hlgA-hlgB-hlgC-sak-scn-seb-sek-seq* | SAMN34573452 |
| 2 | 7-3 | Blood | ST59 | t437 | IVa(2B) | *aph(3')-III-blaZ-erm(B)-mecA-tet(K)* | *aur-hlgA-hlgB-hlgC-sak-scn* | SAMN34573464 |
| 3 | 7-5 | Blood | ST59 | t437 | IVa(2B) | *aph(3')-III-blaZ-erm(B)-mecA-tet(K)* | *aur-hlgA-hlgB-hlgC-sak-scn* | SAMN34573476 |
| 4 | 7-24 | Blood | ST59 | t437 | IVa(2B) | *aph(3')-III-blaZ-erm(B)-mecA-tet(K)* | *aur-hlgA-hlgB-hlgC-sak-scn-seb-sek-seq* | SAMN34573462 |
| 5 | 7-36 | Blood | ST59 | t437 | IVa(2B) | *aph(3')-III-blaZ-erm(B)-mecA-tet(K)* | *aur-hlgA-hlgB-hlgC-sak-scn-seb-sek-seq* | SAMN34573470 |
| 6 | 7-37 | Blood | ST59 | t437 | IVa(2B) | *aph(3')-III-blaZ-erm(B)-mecA-tet(K)* | *aur-hlgA-hlgB-hlgC-sak-scn-seb-sek-seq* | SAMN34573471 |
| 7 | 7-38 | Blood | ST59 | t437 | IVa(2B) | *aph(3')-III-blaZ-erm(B)-mecA-tet(K)* | *aur-hlgA-hlgB-hlgC-sak-scn-seb-sek-seq* | SAMN34573472 |
| 8 | 7-27 | Blood | ST59 | t437 | IVa(2B) | *aph(3')-III-blaZ-erm(B)-erm(C)-mecA-tet(K)* | *aur-hlgA-hlgB-hlgC-sak-scn-seb-sek-seq* | SAMN34573463 |
| 9 | 7-2 | Blood | ST59 | t437 | IVa(2B) | *blaZ-erm(C)-mecA-tet(K)* | *aur-hlgA-hlgB-hlgC-sak-scn-seb-sek-seq* | SAMN34573458 |
| 10 | 7-12 | Blood | ST59 | t437 | IVa(2B) | *blaZ-mecA-tet(K)* | *aur-hlgA-hlgB-hlgC-sak-scn-seb-sek-seq* | SAMN34573454 |
| 11 | 7-20 | Blood | ST59 | t437 | IVa(2B) | *aph(3')-III-blaZ-erm(B)-mecA* | *aur-hlgA-hlgB-hlgC-lukF-PVL-lukS-PVL-sak-scn-sea-seb-sek-seq* | SAMN34573459 |
| 12 | 7-39 | Blood | ST59 | t437 | IVa(2B) | *aph(3')-III-blaZ-erm(B)-erm(C)-mecA-tet(K)* | *aur-hlgA-hlgB-hlgC-sak-scn-seb-sek-seq* | SAMN34573473 |
| 13 | 7-4 | Blood | ST59 | t437 | Vb(5C2&5) | *aph(3')-III-blaZ-erm(B)-erm(C)-mecA-tet(K)* | *aur-hlgA-hlgB-hlgC-lukF-PVL-lukS-PVL-scn-seb-sek-seq* | SAMN34573474 |
| 14 | 7-32 | Blood | ST59 | t437 | Vb(5C2&5) | *blaZ-erm(B)-mecA* | *aur-hlgA-hlgB-hlgC-lukF-PVL-lukS-PVL-scn-seb-sek-seq* | SAMN34573467 |
| 15 | 7-10 | Blood | ST59 | t441 | IVa(2B) | *aph(3')-III-blaZ-erm(B)-grlA-mecA* | *aur-hlgA-hlgB-hlgC-sak-scn* | SAMN34573453 |
| 16 | 7-8 | Blood | ST338 | t437 | Vb(5C2&5) | *aph(3')-III-cat-erm(B)-mecA-tet(K)* | *aur-hlgA-hlgB-hlgC-sak-scn-sea-seb-sek-seq* | SAMN34573479 |
| 17 | 7-18 | Blood | ST398 | t034 | V(5C2) | *blaZ-mecA* | *aur-hlgA-hlgB-hlgC-sak-scn* | SAMN34573456 |
| 18 | 7-34 | Blood | ST398 | t034 | V(5C2) | *blaZ-erm(C)-mecA* | *aur-hlgA-hlgB-hlgC-sak-scn* | SAMN34573468 |
| 19 | 7-21 | Blood | ST5052 | t437 | IVa(2B) | *aph(3')-III-blaZ-cat-erm(B)-mecA* | *aur-hlgA-hlgB-hlgC-sak-scn-seb-sek-seq* | SAMN34573460 |
| 20 | 7-16 | Blood | ST239 | t030 | III(3A) | *aac(6')-aph(2'')-blaZ-gyrA-grlA-mecA-tet(M)* | *aur-hlgA-hlgB-hlgC-lukD-lukE-sak-scn-sea-splA-splB* | SAMN34573455 |
| 21 | 7-19 | Blood | ST239 | t030 | III(3A) | *aac(6')-aph(2'')-blaZ-gyrA-grlA-mecA-tet(M)* | *aur-hlgA-hlgB-hlgC-lukD-lukE-sak-scn-sea-splA-splB* | SAMN34573457 |
| 22 | 7-23 | Blood | ST239 | t030 | III(3A) | *aac(6')-aph(2'')-blaZ-gyrA-grlA-mecA-tet(M)* | *aur-hlgA-hlgB-hlgC-lukD-lukE-sak-scn-sea-splA-splB* | SAMN34573461 |
| 23 | 7-30 | Blood | ST239 | t030 | III(3A) | *aac(6')-aph(2'')-blaZ-dfrB-grlA-gyrA-mecA-tet(M)* | *aur-hlgA-hlgB-hlgC-lukD-lukE-sak-scn-sea-sek-seq-splA-splB* | SAMN34573465 |
| 24 | 7-6 | Blood | ST239 | t459 | III(3A) | *aac(6')-aph(2'')-blaZ-erm(C)-gyrA-grlA-mecA-tet(M)* | *aur-hlgA-hlgB-hlgC-lukD-lukE-sak-scn-sea-sek-seq-splA-splB* | SAMN34573478 |
| 25 | 7-35 | Blood | ST239 | t459 | III(3A) | *aac(6')-aph(2'')-blaZ-erm(C)-gyrA-grlA-mecA-tet(M)* | *aur-hlgA-hlgB-hlgC-lukD-lukE-sak-scn-sea-sek-seq-splA-splB* | SAMN34573469 |
| 26 | 7-40 | Blood | ST239 | t459 | III(3A) | *aac(6')-aph(2'')-blaZ-erm(C)-grlA-gyrA-mecA-tet(M)* | *aur-hlgA-hlgB-hlgC-lukD-lukE-sak-scn-sea-sek-seq-splA-splB* | SAMN34573475 |
| 27 | 7-55 | Blood | ST5 | t002 | V(5C2) | *aph(2'')-Ia-blaZ-erm(B)-grlA-mecA* | *aur-hlgA-hlgB-hlgC-lukD-lukE-sak-scn-sec-sed-seg-sei-sej-sel-sem-sen-seo-ser-seu-splA-splB* | SAMN34573477 |
| 28 | 7-31 | Blood | ST88 | t2310 | IVc(2B) | *blaZ-erm(C)-mecA* | *aur-hlgA-hlgB-hlgC-lukD-lukE-sak-scn-sep-splA-splB* | SAMN34573466 |
| 29 | 7-13 | Cerebrospinal fluid | ST239 | t030 | III(3A) | *aac(6')-aph(2'')-blaZ-gyrA-grlA-mecA-tet(M)* | *aur-hlgA-hlgB-hlgC-lukD-lukE-sak-scn-sea-splA-splB* | SAMN34573451 |
| 30 | S2 | Secretion | ST59 | t437 | IVa(2B) | *aph(3')-III-blaZ-erm(B)-mecA-tet(K)* | *aur-hlgA-hlgB-hlgC-sak-scn-seb-sek-seq* | SAMN34573401 |
| 31 | S7 | Secretion | ST59 | t437 | IVa(2B) | *aph(3')-III-erm(B)-mecA* | *aur-hlgA-hlgB-hlgC-sak-scn-seb-sek-seq* | SAMN34573413 |
| 32 | S21 | Secretion | ST59 | t437 | IVa(2B) | *aph(3')-III-erm(B)-mecA* | *aur-hlgA-hlgB-hlgC-sak-scn-seb-sek-seq* | SAMN34573403 |
| 33 | S27 | Secretion | ST59 | t437 | IVa(2B) | *aph(3')-III-erm(B)-mecA* | *aur-hlgA-hlgB-hlgC-sak-scn-seb-sek-seq* | SAMN34573406 |
| 34 | S31 | Secretion | ST59 | t437 | IVa(2B) | *aph(3')-III-blaZ-erm(B)-mecA-tet(K)* | *aur-hlgA-hlgB-hlgC-sak-scn-seb-sek-seq* | SAMN34573409 |
| 35 | S33 | Secretion | ST59 | t437 | IVa(2B) | *aph(3')-III-blaZ-erm(B)-erm(C)-mecA-tet(K)* | *aur-hlgA-hlgB-hlgC-sak-scn-seb-sek-seq* | SAMN34573410 |
| 36 | Y3 | Secretion | ST59 | t437 | IVa(2B) | *blaZ-mecA* | *aur-hlgA-hlgB-hlgC-lukF-PVL-lukS-PVL-scn* | SAMN34573417 |
| 37 | Y6 | Secretion | ST59 | t437 | IVa(2B) | *mecA* | *aur-hlgA-hlgB-hlgC-sak-scn-seb-sek-seq* | SAMN34573418 |
| 38 | Y7 | Secretion | ST59 | t437 | IVa(2B) | *aph(3')-III-blaZ-erm(B)-mecA* | *aur-hlgA-hlgB-hlgC-sak-scn* | SAMN34573419 |
| 39 | Y12 | Secretion | ST59 | t437 | IVa(2B) | *aph(3')-III-blaZ-erm(B)-mecA* | *aur-hlgA-hlgB-hlgC-sak-scn-seb-sek-seq* | SAMN34573493 |
| 40 | Y13 | Secretion | ST59 | t437 | IVa(2B) | *mecA* | *aur-hlgA-hlgB-hlgC-lukF-PVL-lukS-PVL-scn-seb-sek-seq* | SAMN34573416 |
| 41 | S6 | Secretion | ST59 | t441 | IVa(2B) | *aph(3')-III-blaZ-erm(B)-grlA-mecA-tet(K)* | *aur-hlgA-hlgB-hlgC-sak-scn-seb-sek-seq* | SAMN34573412 |
| 42 | S29 | Secretion | ST59 | t441 | IVa(2B) | *aph(3')-III-blaZ-erm(B)-mecA* | *aur-hlgA-hlgB-hlgC-sak-scn* | SAMN34573407 |
| 43 | S20 | Secretion | ST59 | t13774 | IVa(2B) | *aph(3')-III-blaZ-erm(B)-erm(C)-mecA* | *aur-hlgA-hlgB-hlgC-sak-scn-seb-sek-seq* | SAMN34573402 |
| 44 | S4 | Secretion | ST509 | t375 | IVa(2B) | *blaZ-dfrG-mecA* | *aur-edinC-etb-hlgA-hlgB-hlgC-sak-scn-sem-seo* | SAMN34573411 |
| 45 | Y1 | Secretion | ST239 | t459 | III(3A) | *aac(6')-aph(2'')-blaZ-erm(C)-gyrA-grlA-mecA-tet(M)* | *aur-hlgA-hlgB-hlgC-lukD-lukE-sak-scn-sea-sek-seq-splA-splB* | SAMN34573415 |
| 46 | S25 | Secretion | ST398 | t571 | III(3A) | *blaZ-erm(T)-mecA* | *aur-hlgA-hlgB-hlgC-sak-scn* | SAMN34573405 |
| 47 | S23 | Secretion | ST398 | t1928 | V(5C2) | *blaZ-mecA* | *aur-hlgA-hlgB-hlgC-sak-scn* | SAMN34573404 |
| 48 | S30 | Secretion | ST88 | t2310 | IVc(2B) | *blaZ-erm(C)-mecA* | *aur-hlgA-hlgB-hlgC-lukD-lukE-sak-scn-sep-splA-splB* | SAMN34573408 |
| 49 | S8 | Secretion | ST9 | t899 | XII(9C2) | *aac(6')-aph(2'')-aadD-blaZ-dfrG-erm(C)-fexA-gyrA-grlA-lsa(E)-lnu(B)-mecA-tet(L)* | *aur-hlgA-hlgB-hlgC-seo* | SAMN34573414 |
| 50 | 5-2 | Fecal | ST59 | t437 | IVa(2B) | *aph(3')-III-blaZ-erm(B) -mecA* | *aur-hlgA-hlgB-hlgC-sak-scn-seb-sek-seq* | SAMN34573427 |
| 51 | 5-6 | Fecal | ST59 | t437 | IVa(2B) | *aph(3')-III-erm(B)-mecA* | *aur-hlgA-hlgB-hlgC-sak-scn-seb-sek-seq* | SAMN34573436 |
| 52 | 5-12 | Fecal | ST59 | t437 | IVa(2B) | *aph(3')-III-blaZ-erm(B)-mecA-tet(K)* | *aur-hlgA-hlgB-hlgC-sak-scn-seb-sek-seq* | SAMN34573420 |
| 53 | 5-16 | Fecal | ST59 | t437 | IVa(2B) | *aph(3')-III-blaZ-erm(B) -mecA* | *aur-hlgA-hlgB-hlgC-sak-scn-seb-sek-seq* | SAMN34573424 |
| 54 | 5-17 | Fecal | ST59 | t437 | IVa(2B) | *aph(3')-III-blaZ-erm(B) -mecA* | *aur-hlgA-hlgB-hlgC-sak-scn-seb-sek-seq* | SAMN34573425 |
| 55 | 5-19 | Fecal | ST59 | t437 | IVa(2B) | *aph(3')-III-blaZ-erm(B) -mecA* | *aur-hlgA-hlgB-hlgC-lukF-PVL-lukS-PVL-scn-seb-sek-seq* | SAMN34573426 |
| 56 | 5-22 | Fecal | ST59 | t437 | IVa(2B) | *blaZ-erm(C)-mecA-tet(K)* | *aur-hlgA-hlgB-hlgC-sak-scn-seb-sek-seq* | SAMN34573428 |
| 57 | 5-23 | Fecal | ST59 | t437 | IVa(2B) | *aph(3')-III-blaZ-erm(B) -mecA* | *aur-hlgA-hlgB-hlgC-lukF-PVL-lukS-PVL-sak-scn-sea-seb-sek-seq* | SAMN34573429 |
| 58 | 5-25 | Fecal | ST59 | t437 | IVa(2B) | *blaZ-erm(B)-mecA* | *aur-hlgA-hlgB-hlgC-sak-scn-seb-sek-seq* | SAMN34573430 |
| 59 | 5-27 | Fecal | ST59 | t437 | IVa(2B) | *blaZ-erm(B)-mecA* | *aur-hlgA-hlgB-hlgC-sak-scn-seb-sek-seq* | SAMN34573431 |
| 60 | 5-30 | Fecal | ST59 | t437 | IVa(2B) | *blaZ-erm(C)-mecA-tet(K)* | *aur-hlgA-hlgB-hlgC-sak-scn-seb-sek-seq* | SAMN34573433 |
| 61 | 5-31 | Fecal | ST59 | t437 | IVa(2B) | *blaZ-mecA* | *aur-hlgA-hlgB-hlgC-lukF-PVL-lukS-PVL-scn-seb-sek-seq* | SAMN34573434 |
| 62 | 5-39 | Fecal | ST59 | t437 | IVa(2B) | *aph(3')-III-blaZ-erm(B) -mecA* | *aur-hlgA-hlgB-hlgC-sak-scn-seb-sek-seq* | SAMN34573435 |
| 63 | 5-13 | Fecal | ST59 | t441 | IVa(2B) | *aph(3')-III-erm(B)-mecA* | *aur-hlgA-hlgB-hlgC-sak-scn-seb-sek-seq* | SAMN34573421 |
| 64 | 5-29 | Fecal | ST59 | t441 | IVa(2B) | *aph(3')-III-blaZ-erm(B)-mecA-tet(K)* | *aur-hlgA-hlgB-hlgC-sak-scn-seb-sek-seq* | SAMN34573432 |
| 65 | 5-9 | Fecal | ST6576 | t701 | IVd(2B) | *blaZ-mecA* | *aur-hlgA-hlgB-hlgC-lukD-lukE-sak-scn-sea-splA-splB-splE* | SAMN34573437 |
| 66 | 5-15 | Fecal | ST6576 | t701 | IVd(2B) | *blaZ-mecA* | *aur-hlgA-hlgB-hlgC-lukD-lukE-sak-scn-sea-splA-splB-splE* | SAMN34573423 |
| 67 | 5-14 | Fecal | ST45 | t116 | IVa(2B) | *blaZ-mecA* | *aur-hlgA-hlgB-hlgC-sec-seg-sei-sel-sem-sen-seo-seu* | SAMN34573422 |
| 68 | 1-7 | Chicken | ST59 | t437 | IVa(2B) | *blaZ-erm(C)-mecA* | *aur-hlgA-hlgB-hlgC-sak-scn-seb-sek-seq* | SAMN34573441 |
| 69 | 1-25 | Chicken | ST59 | t437 | IVa(2B) | *aph(3')-III-blaZ-erm(B)-mecA-tet(K)* | *aur-hlgA-hlgB-hlgC-sak-scn-seb-sek-seq* | SAMN34573439 |
| 70 | 2-6 | Chicken | ST59 | t441 | IVa(2B) | *erm(C)-mecA* | *aur-hlgA-hlgB-hlgC-sak-scn-seb-sek-seq* | SAMN34573445 |
| 71 | 2-32 | Chicken | ST59 | t8391 | IVa(2B) | *aph(3')-III-blaZ-erm(B) -mecA* | *aur-hlgA-hlgB-hlgC-sak-scn-seb-sek-seq* | SAMN34573443 |
| 72 | 2-39 | Chicken | ST59 | t4193 | IVa(2B) | *blaZ-erm(C)-gyrA-grlA-mecA* | *aur-hlgA-hlgB-hlgC-sak-scn-sea* | SAMN34573444 |
| 73 | 3-16 | Chicken | ST59 | t3515 | IVg(2B) | *blaZ-cat-erm(B)-mecA-tet(K)* | *aur-hlgA-hlgB-hlgC-sak-scn-seb-sek-seq* | SAMN34573446 |
| 74 | 1-6 | Chicken | ST9 | t899 | XII(9C2) | *aadD-aac(6')-aph(2'')-blaZ-dfrG-erm(C)-fexA-gyrA-grlA-lnu(B)-lsa(E)-mecA-tet(L)* | *aur-hlgA-hlgB-hlgC-seg-sei-sem-sen-seo-seu* | SAMN34573440 |
| 75 | 1-19 | Chicken | ST9 | t899 | XII(9C2) | *aadD-aac(6')-aph(2'')-blaZ-dfrG-erm(C)-fexA-gyrA-grlA-lnu(B)-lsa(E)-mecA-tet(L)* | *aur-hlgA-hlgB-hlgC-seg-sei-sem-sen-seo-seu* | SAMN34573438 |
| 76 | 2-12 | Chicken | ST9 | t899 | XII(9C2) | *aadD-aac(6')-aph(2'')-blaZ-dfrG-erm(C)-fexA-gyrA-grlA-lsa(E)-lnu(B)-mecA-tet(L)* | *aur-hlgA-hlgB-hlgC-seg-sei-sem-sen-seo-seu* | SAMN34573442 |
| 77 | 3-5 | Chicken | ST9 | t899 | XII(9C2) | *aac(6')-aph(2'')-aadD-blaZ-dfrG-erm(C)-gyrA-grlA-lnu(B)-lsa(E)-mecA-tet(L)* | *aur-hlgA-hlgB-hlgC-seg-sei-sem-sen-seo-seu* | SAMN34573447 |
| 78 | 4-3 | Chicken | ST9 | t899 | XII(9C2) | *aac(6')-aph(2'')-aadD-blaZ-dfrG-erm(C)-fexA-grlA-gyrA-lnu(B)-lsa(E)-mecA-tet(L)* | *aur-hlgA-hlgB-hlgC-seg-sei-sem-sen-seo-seu* | SAMN34573449 |
| 79 | 4-10 | Chicken | ST9 | t899 | XII(9C2) | *aadD-aac(6')-aph(2'')-blaZ-dfrG-erm(C)-fexA-grlA-gyrA-lsa(E)-lnu(B)-mecA-tet(L)* | *aur-hlgA-hlgB-hlgC-seg-sei-sem-sen-seo-seu* | SAMN34573448 |
| 80 | 4-9 | Chicken | ST398 | t011 | Vc(5C2&5) | *blaZ-dfrG-erm(B)-lnu(B)-lsa(E)-mecA-tet(K)-tet(M)* | *aur-hlgA-hlgB-hlgC* | SAMN34573450 |
| 81 | 1-22 | Pork | ST59 | t437 | IVa(2B) | *aph(3')-III-blaZ-erm(B)-mecA* | *aur-hlgA-hlgB-hlgC-sak-scn-seb-sek-seq* | SAMN34573480 |
| 82 | 2884 | Pork | ST59 | t437 | IVa(2B) | *aph(3')-III-blaZ-erm(B)-mecA-tet(K)* | *aur-hlgA-hlgB-hlgC-sak-scn-seb-sek-seq* | SAMN34573482 |
| 83 | 2885 | Pork | ST59 | t437 | IVa(2B) | *aph(3')-III-blaZ-erm(B)-mecA-tet(K)* | *aur-hlgA-hlgB-hlgC-sak-scn-seb-sek-seq* | SAMN34573483 |
| 84 | 3115 | Pork | ST59 | t437 | IVa(2B) | *aph(3')-III-blaZ-erm(B)-erm(C)-mecA-tet(K)* | *aur-hlgA-hlgB-hlgC-sak-scn-seb-sek-seq* | SAMN34573488 |
| 85 | 3116 | Pork | ST59 | t437 | IVa(2B) | *aph(3')-III-blaZ-erm(B)-mecA-tet(K)* | *aur-hlgA-hlgB-hlgC-sak-scn-seb-sek-seq* | SAMN34573489 |
| 86 | 3117 | Pork | ST59 | t437 | IVa(2B) | *aph(3')-III-blaZ-erm(B)-mecA-tet(K)* | *aur-hlgA-hlgB-hlgC-sak-scn-seb-sek-seq* | SAMN34573490 |
| 87 | 3118 | Pork | ST59 | t437 | IVa(2B) | *aph(3')-III-blaZ-erm(B)-erm(C)-mecA-tet(K)* | *aur-hlgA-hlgB-hlgC-sak-scn-seb-sek-seq* | SAMN34573491 |
| 88 | 2886 | Pork | ST9 | t899 | XII(9C2) | *aac(6')-aph(2'') -aadD-blaZ-dfrG-erm(C)-fexA-grlA-gyrA-lnu(B)-lsa(E)-mecA-tet(L)* | *aur-hlgA-hlgB-hlgC-seg-sei-sem-sen-seo-seu* | SAMN34573484 |
| 89 | 2887 | Pork | ST9 | t899 | XII(9C2) | *aac(6')-aph(2'')-aadD-blaZ-dfrG-erm(C)-gyrA-grlA-lnu(B)-lsa(E)-mecA-tet(L)* | *aur-hlgA-hlgB-hlgC-seg-sei-sem-sen-seo-seu* | SAMN34573485 |
| 90 | 2888 | Pork | ST9 | t899 | XII(9C2) | *aac(6')-aph(2'')-aadD-blaZ-cfr-dfrG-erm(C)-fexA-gyrA-grlA-lsa(E)-lnu(B)-mecA-tet(L)* | *aur-hlgA-hlgB-hlgC-seo* | SAMN34573486 |
| 91 | 2-14 | Pork | ST398 | t011 | Vc(5C2&5) | *blaZ-dfrG-lnu(B)-lsa(E)-mecA-tet(M)-tet(K)* | *aur-hlgA-hlgB-hlgC* | SAMN34573481 |
| 92 | 4-21 | Pork | ST398 | t011 | Vc(5C2&5) | *blaZ-dfrG-erm(B)-lnu(B)-lsa(E)-mecA-tet(K)-tet(M)* | *aur-hlgA-hlgB-hlgC* | SAMN34573492 |
| 93 | 3114 | Pork | ST88 | t2788 | IVa(2B) | *blaZ-mecA* | *aur-hlgA-hlgB-hlgC-lukD-lukE-sak-scn-sep-splA-splB* | SAMN34573487 |
