# Supplementary material for: Genome-based surveillance reveals cross-transmission of MRSA ST59 between humans and retail livestock products in Hanzhong, China
Source: Front Microbiol. 2024 Apr 29;15:1392134. doi: 10.3389/fmicb.2024.1392134 (PMC11089119; doi:10.3389/fmicb.2024.1392134)
Supplement: Supplementary file 2 [file Table_2.docx]

**Supplementary Table 2 Minimal inhibitory concentrations (MICs) and antimicrobial resistant phenotypes of food and patient MRSA isolates from Hanzhong, China**

|  | Strains | GEN | ERY | TET | LEV | CLI | SXT | RIF | Resistant phenotypes |
| --- | --- | --- | --- | --- | --- | --- | --- | --- | --- |
| 1 | 7-1 | 0.5 | ＞16 | 16 | 0.25 | ＞8 | ≤0.25/4.75 | 0.125 | CLI-ERY-TET |
| 2 | 7-2 | 0.5 | ＞16 | ＞16 | 0.25 | ≤0.125 | ≤0.25/4.75 | ≤0.06 | ERY-TET |
| 3 | 7-3 | 0.5 | ＞16 | 16 | 0.25 | ＞8 | ≤0.25/4.75 | ≤0.06 | CLI-ERY-TET |
| 4 | 7-4 | 0.5 | ＞16 | 8 | ≤0.125 | ＞8 | ≤0.25/4.75 | ≤0.06 | CLI-ERY |
| 5 | 7-5 | 0.5 | ＞16 | 16 | 0.25 | ＞8 | ≤0.25/4.75 | ≤0.06 | CLI-ERY-TET |
| 6 | 7-10 | 0.5 | ＞16 | 0.5 | 2 | ＞8 | ≤0.25/4.75 | ≤0.06 | CLI-ERY |
| 7 | 7-12 | 0.5 | 0.5 | 16 | 0.25 | ≤0.125 | ≤0.25/4.75 | ≤0.06 | TET |
| 8 | 7-20 | 0.25 | ＞16 | 0.5 | 0.25 | ＞8 | ≤0.25/4.75 | ≤0.06 | CLI-ERY |
| 9 | 7-24 | ≤0.25 | ＞16 | 8 | 0.25 | ＞8 | ≤0.25/4.75 | ≤0.06 | CLI-ERY |
| 10 | 7-27 | 0.5 | ＞16 | 16 | 0.25 | ＞8 | ≤0.25/4.75 | ≤0.06 | CLI-ERY-TET |
| 11 | 7-32 | 0.5 | ＞16 | 0.5 | 0.25 | ＞8 | ≤0.25/4.75 | ≤0.06 | CLI-ERY |
| 12 | 7-36 | 0.5 | ＞16 | 8 | 0.25 | ＞8 | ≤0.25/4.75 | ≤0.06 | CLI-ERY |
| 13 | 7-37 | ≤0.25 | ＞16 | 16 | ≤0.125 | ＞8 | ≤0.25/4.75 | ≤0.06 | CLI-ERY-TET |
| 14 | 7-38 | 0.5 | ＞16 | 0.5 | 0.25 | ＞8 | ≤0.25/4.75 | ≤0.06 | CLI-ERY |
| 15 | 7-39 | 0.5 | ＞16 | 16 | 0.25 | ＞8 | ≤0.25/4.75 | ≤0.06 | CLI-ERY-TET |
| 16 | 7-6 | ＞16 | ＞16 | ＞16 | ＞8 | ＞8 | ≤0.25/4.75 | ＞4 | CLI-ERY-GEN-LEV-TET-RIF |
| 17 | 7-16 | ＞16 | ＞16 | ＞16 | ＞8 | ≤0.125 | ≤0.25/4.75 | ＞4 | ERY-GEN-LEV-RIF-TET |
| 18 | 7-19 | ＞16 | ＞16 | ＞16 | ＞8 | ≤0.125 | ≤0.25/4.75 | ＞4 | ERY-GEN-LEV-RIF-TET |
| 19 | 7-23 | ＞16 | 4 | ＞16 | ＞8 | ≤0.125 | ≤0.25/4.75 | ＞4 | GEN-LEV-RIF-TET |
| 20 | 7-30 | ＞16 | 0.5 | ＞16 | ＞8 | ≤0.125 | 1/19 | ＞4 | GEN-LEV-RIF-TET |
| 21 | 7-35 | ＞16 | ＞16 | ＞16 | ＞8 | ＞8 | ≤0.25/4.75 | ＞4 | CLI-ERY-GEN-LEV-TET-RIF |
| 22 | 7-40 | ＞16 | ＞16 | ＞16 | ＞8 | ＞8 | ≤0.25/4.75 | ＞4 | CLI-ERY-GEN-LEV-TET-RIF |
| 23 | 7-18 | 0.5 | 0.5 | 0.5 | 1 | ≤0.125 | ≤0.25/4.75 | ≤0.06 | -* |
| 24 | 7-34 | 0.5 | ＞16 | 0.5 | 0.25 | ≤0.125 | ≤0.25/4.75 | ≤0.06 | ERY |
| 25 | 7-8 | 0.5 | ＞16 | 16 | 0.25 | ＞8 | ≤0.25/4.75 | ≤0.06 | CLI-ERY-TET |
| 26 | 7-55 | ＞16 | ＞16 | 0.5 | 1 | ＞8 | ＞8/152 | ≤0.06 | CLI-ERY-GEN-SXT |
| 27 | 7-21 | 0.5 | ＞16 | ≤0.25 | 0.25 | ＞8 | ≤0.25/4.75 | ≤0.06 | CLI-ERY |
| 28 | 7-31 | ≤0.25 | ＞16 | 0.5 | 0.25 | ≤0.125 | ≤0.25/4.75 | ≤0.06 | ERY |
| 29 | S2 | 0.5 | ＞16 | 16 | 0.25 | ＞8 | ≤0.25/4.75 | ≤0.06 | CLI-ERY-TET |
| 30 | S6 | 0.5 | ＞16 | 16 | 0.5 | ＞8 | ≤0.25/4.75 | ≤0.06 | CLI-ERY-TET |
| 31 | S7 | 0.5 | ＞16 | ≤0.25 | 0.25 | ＞8 | ≤0.25/4.75 | ≤0.06 | CLI-ERY |
| 32 | S20 | 0.5 | ＞16 | ≤0.25 | 0.25 | ＞8 | ≤0.25/4.75 | ≤0.06 | CLI-ERY |
| 33 | S21 | 0.5 | ＞16 | 0.5 | 0.25 | ＞8 | ≤0.25/4.75 | ≤0.06 | CLI-ERY |
| 34 | S27 | 0.5 | ＞16 | 0.5 | 0.25 | ＞8 | ≤0.25/4.75 | ≤0.06 | CLI-ERY |
| 35 | S29 | 0.5 | ＞16 | 0.5 | 0.25 | ＞8 | ≤0.25/4.75 | ≤0.06 | CLI-ERY |
| 36 | S31 | 0.5 | ＞16 | ＞16 | 0.25 | ＞8 | ≤0.25/4.75 | ≤0.06 | CLI-ERY-TET |
| 37 | S33 | 0.5 | ＞16 | ＞16 | 0.25 | ＞8 | ≤0.25/4.75 | ≤0.06 | CLI-ERY-TET |
| 38 | Y3 | ＞16 | ＞16 | ＞16 | ＞8 | ＞8 | ≤0.25/4.75 | ＞4 | CLI-ERY-GEN-LEV-RIF-TET |
| 39 | Y6 | 0.5 | ≤0.25 | ≤0.25 | 0.25 | ≤0.125 | ≤0.25/4.75 | ≤0.06 | - |
| 40 | Y7 | 0.5 | ＞16 | ≤0.25 | 0.25 | ＞8 | ≤0.25/4.75 | ≤0.06 | CLI-ERY |
| 41 | Y12 | 0.5 | ＞16 | ≤0.25 | 0.25 | ＞8 | ≤0.25/4.75 | ≤0.06 | CLI-ERY |
| 42 | Y13 | 0.5 | 0.5 | 0.5 | 0.25 | ≤0.125 | ≤0.25/4.75 | ≤0.06 | - |
| 43 | S23 | 0.5 | 0.5 | 0.5 | 0.25 | ≤0.125 | ≤0.25/4.75 | ≤0.06 | - |
| 44 | S25 | 0.5 | ＞16 | 0.5 | 0.5 | ＞8 | ≤0.25/4.75 | ≤0.06 | CLI-ERY |
| 45 | Y1 | ＞16 | ＞16 | ＞16 | ＞8 | ＞8 | ≤0.25/4.75 | ＞4 | CLI-ERY-GEN-LEV-RIF-TET |
| 46 | S4 | 0.5 | ≤0.25 | ≤0.25 | 0.5 | ≤0.125 | 1/19 | 2 | - |
| 47 | S30 | 0.5 | ＞16 | 0.5 | 0.25 | ≤0.125 | ≤0.25/4.75 | ≤0.06 | ERY |
| 48 | S8 | ＞16 | ＞16 | ＞16 | 2 | ＞8 | 1/19 | ≤0.06 | CLI-ERY-GEN-TET |
| 49 | 5-2 | ≤0.25 | ＞16 | ≤0.25 | 0.25 | ＞8 | ≤0.25/4.75 | ≤0.06 | CLI-ERY |
| 50 | 5-6 | ≤0.25 | ＞16 | ≤0.25 | 0.25 | ＞8 | ≤0.25/4.75 | ≤0.06 | CLI-ERY |
| 51 | 5-12 | 0.5 | ＞16 | 8 | ≤0.125 | ＞8 | ≤0.25/4.75 | ≤0.06 | CLI-ERY |
| 52 | 5-13 | ≤0.25 | ＞16 | ≤0.25 | ≤0.125 | ＞8 | ≤0.25/4.75 | ≤0.06 | CLI-ERY |
| 53 | 5-16 | 0.5 | ＞16 | 0.5 | 0.25 | ＞8 | ≤0.25/4.75 | ≤0.06 | CLI-ERY |
| 54 | 5-17 | 0.5 | ＞16 | ≤0.25 | 0.25 | ＞8 | ≤0.25/4.75 | ≤0.06 | CLI-ERY |
| 55 | 5-19 | 0.5 | ＞16 | ≤0.25 | 0.25 | ＞8 | ≤0.25/4.75 | ≤0.06 | CLI-ERY |
| 56 | 5-22 | 0.5 | ＞16 | ＞16 | 0.25 | ≤0.125 | ≤0.25/4.75 | ≤0.06 | ERY-TET |
| 57 | 5-23 | 0.5 | ＞16 | ≤0.25 | ≤0.125 | ＞8 | ≤0.25/4.75 | ≤0.06 | CLI-ERY |
| 58 | 5-25 | 0.5 | ＞16 | 0.5 | 0.25 | ＞8 | ≤0.25/4.75 | ≤0.06 | CLI-ERY |
| 59 | 5-27 | 0.5 | ＞16 | ≤0.25 | 0.25 | ＞8 | ≤0.25/4.75 | 0.125 | CLI-ERY |
| 60 | 5-29 | 0.5 | ＞16 | 16 | 0.25 | ＞8 | ≤0.25/4.75 | 0.125 | CLI-ERY-TET |
| 61 | 5-30 | 0.5 | ＞16 | 16 | 0.25 | ≤0.125 | 0.5/9.5 | ≤0.06 | ERY-TET |
| 62 | 5-31 | ≤0.25 | ＞16 | 8 | 0.5 | ＞8 | ≤0.25/4.75 | 0.125 | CLI-ERY |
| 63 | 5-39 | 0.5 | ＞16 | ≤0.25 | 0.25 | ＞8 | ≤0.25/4.75 | ≤0.06 | CLI-ERY |
| 64 | 5-9 | ≤0.25 | ≤0.25 | ≤0.25 | 0.25 | ≤0.125 | ≤0.25/4.75 | ≤0.06 | - |
| 65 | 5-15 | 0.5 | ≤0.25 | 0.5 | 0.25 | ≤0.125 | ≤0.25/4.75 | ≤0.06 | - |
| 66 | 5-14 | ≤0.25 | 0.5 | ≤0.25 | ≤0.125 | ≤0.125 | ≤0.25/4.75 | 2 | - |
| 67 | 7-13 | ＞16 | ＞16 | ＞16 | ＞8 | ≤0.125 | ≤0.25/4.75 | ＞4 | ERY-GEN-LEV-RIF-TET |
| 68 | 1-7 | 0.5 | ＞16 | ≤0.25 | 0.25 | ≤0.125 | ≤0.25/4.75 | ≤0.06 | ERY |
| 69 | 1-25 | 0.5 | ＞16 | 16 | 0.25 | ＞8 | ≤0.25/4.75 | ≤0.06 | CLI-ERY-TET |
| 70 | 2-6 | 0.5 | ＞16 | 0.5 | 0.25 | ≤0.125 | 0.5/9.5 | ≤0.06 | ERY |
| 71 | 2-32 | 0.5 | ＞16 | ≤0.25 | 0.25 | ＞8 | ≤0.25/4.75 | ≤0.06 | CLI-ERY |
| 72 | 2-39 | 0.5 | ＞16 | ≤0.25 | 8 | ＞8 | 0.5/9.5 | ≤0.06 | CLI-ERY-LEV |
| 73 | 3-16 | 0.5 | ＞16 | 16 | ≤0.125 | ＞8 | ≤0.25/4.75 | ≤0.06 | CLI-ERY-TET |
| 74 | 1-6 | ＞16 | ＞16 | ＞16 | 4 | ＞8 | ＞8/152 | ≤0.06 | CLI-ERY-GEN-LEV-SXT-TET |
| 75 | 1-19 | ＞16 | ＞16 | ＞16 | 4 | ＞8 | ＞8/152 | ≤0.06 | CLI-ERY-GEN-LEV-SXT-TET |
| 76 | 2-12 | ＞16 | ＞16 | ＞16 | 2 | ＞8 | ＞8/152 | ≤0.06 | CLI-ERY-GEN-SXT-TET |
| 77 | 3-5 | ＞16 | ＞16 | ＞16 | 2 | ＞8 | ＞8/152 | 2 | CLI-ERY-GEN-SXT-TET |
| 78 | 4-3 | ＞16 | ＞16 | ＞16 | 2 | ＞8 | ＞8/152 | ≤0.06 | CLI-ERY-GEN-SXT-TET |
| 79 | 4-10 | ＞16 | ＞16 | ＞16 | 2 | ＞8 | ＞8/152 | ≤0.06 | CLI-ERY-GEN-SXT-TET |
| 80 | 4-9 | 1 | ＞16 | ＞16 | 2 | ＞8 | 0.5/9.5 | ≤0.06 | CLI-ERY-TET |
| 81 | 1-22 | 0.5 | ＞16 | ≤0.25 | 0.25 | ＞8 | ≤0.25/4.75 | ≤0.06 | CLI-ERY |
| 82 | 2884 | 0.5 | ＞16 | 16 | 0.5 | ＞8 | ≤0.25/4.75 | ≤0.06 | CLI-ERY-TET |
| 83 | 2885 | ≤0.25 | ＞16 | 16 | 0.5 | ＞8 | ≤0.25/4.75 | ≤0.06 | CLI-ERY-TET |
| 84 | 3115 | ≤0.25 | ＞16 | 16 | 0.5 | ＞8 | ≤0.25/4.75 | ≤0.06 | CLI-ERY-TET |
| 85 | 3116 | ≤0.25 | ＞16 | 16 | 0.5 | ＞8 | ≤0.25/4.75 | ≤0.06 | CLI-ERY-TET |
| 86 | 3117 | 0.5 | ＞16 | 16 | 0.5 | ＞8 | ≤0.25/4.75 | ≤0.06 | CLI-ERY-TET |
| 87 | 3118 | 1 | ＞16 | 16 | 0.5 | ＞8 | ≤0.25/4.75 | ≤0.06 | CLI-ERY-TET |
| 88 | 2886 | ＞16 | ＞16 | ＞16 | 4 | ＞8 | ＞8/152 | 2 | CLI-ERY-GEN-LEV-SXT-TET |
| 89 | 2887 | ＞16 | ＞16 | ＞16 | 4 | ＞8 | ＞8/152 | ≤0.06 | CLI-ERY-GEN-LEV-SXT-TET |
| 90 | 2888 | ＞16 | ＞16 | ＞16 | 4 | ＞8 | ＞8/152 | ≤0.06 | CLI-ERY-GEN-LEV-SXT-TET |
| 91 | 2-14 | 0.5 | ≤0.25 | ＞16 | ≤0.125 | 4 | ≤0.25/4.75 | ≤0.06 | CLI-TET |
| 92 | 4-21 | 0.5 | ＞16 | ＞16 | 0.25 | ＞8 | ≤0.25/4.75 | ≤0.06 | CLI-ERY-TET |
| 93 | 3114 | 0.5 | 0.5 | ≤0.25 | 0.25 | ≤0.125 | ≤0.25/4.75 | ≤0.06 | - |

*: GEN, Gentamicin; ERY, Erythromycin; CLI, Clindamycin; LEV, Levofloxacin; SXT, Trimethoprim-sulfamethoxazole; RIF, Rifampin; TET, Tetracycline.
