# Supplementary material for: Genome-based surveillance reveals cross-transmission of MRSA ST59 between humans and retail livestock products in Hanzhong, China
Source: Front Microbiol. 2024 Apr 29;15:1392134. doi: 10.3389/fmicb.2024.1392134 (PMC11089119; doi:10.3389/fmicb.2024.1392134)
Supplement: Supplementary file 3 [file Table_3.doc]

**Supplementary Table 3 Summary of sequencing data quality of food and patient MRSA isolates from Hanzhong, China**

|  | **Strain** | **Total Length(bp)** | **Number of contigs** | **N50 Length(bp)** | **Min Length(bp)** | **Max Length(bp)** | **Sequence GC(%)** |
| --- | --- | --- | --- | --- | --- | --- | --- |
| 1 | 7-1 | 27,72,024 | 50 | 153,998 | 327 | 452,738 | 33.19 |
| 2 | 7-2 | 2,800,515 | 54 | 168,178 | 327 | 452,730 | 33.13 |
| 3 | 7-3 | 2,753,540 | 52 | 147,736 | 327 | 449,880 | 33.20 |
| 4 | 7-4 | 2,829,964 | 66 | 147,603 | 317 | 349,771 | 33.00 |
| 5 | 7-5 | 2,759,831 | 54 | 149,343 | 327 | 452,617 | 32.96 |
| 6 | 7-10 | 2,753,387 | 46 | 168,164 | 327 | 451,926 | 33.05 |
| 7 | 7-12 | 2,754,564 | 49 | 153,662 | 327 | 450,051 | 33.06 |
| 8 | 7-20 | 2,770,514 | 69 | 147,687 | 316 | 353,342 | 33.16 |
| 9 | 7-24 | 2,817,969 | 54 | 147,626 | 327 | 452,790 | 33.11 |
| 10 | 7-27 | 2,815,115 | 60 | 149,285 | 317 | 452,713 | 33.06 |
| 11 | 7-32 | 2,772,110 | 54 | 153,849 | 304 | 355,130 | 33.07 |
| 12 | 7-36 | 2,813,706 | 67 | 149,165 | 317 | 452,712 | 33.20 |
| 13 | 7-37 | 2,829,896 | 65 | 149,343 | 305 | 452,765 | 33.65 |
| 14 | 7-38 | 2,836,138 | 68 | 151,564 | 317 | 452,738 | 33.20 |
| 15 | 7-39 | 2,771,871 | 53 | 149,344 | 317 | 452,411 | 33.12 |
| 16 | 7-6 | 2,838,752 | 64 | 105,783 | 327 | 315,558 | 33.10 |
| 17 | 7-16 | 2,824,068 | 61 | 113,934 | 403 | 248,173 | 33.08 |
| 18 | 7-19 | 2,869,344 | 63 | 111,922 | 403 | 248,173 | 33.26 |
| 19 | 7-23 | 2,877,185 | 70 | 93,292 | 327 | 313,758 | 33.11 |
| 20 | 7-30 | 2,848,438 | 71 | 109,356 | 327 | 315,664 | 33.19 |
| 21 | 7-35 | 2,887,719 | 65 | 109,460 | 308 | 311,013 | 33.17 |
| 22 | 7-40 | 2,844,781 | 62 | 109,460 | 327 | 315,559 | 33.12 |
| 23 | 7-18 | 2,710,774 | 46 | 340,741 | 305 | 564,760 | 33.10 |
| 24 | 7-34 | 2,712,305 | 40 | 340,741 | 307 | 564,761 | 33.16 |
| 25 | 7-8 | 2,870,646 | 98 | 97,021 | 317 | 452,683 | 33.02 |
| 26 | 7-55 | 2,804,572 | 31 | 324,959 | 317 | 680,224 | 33.39 |
| 27 | 7-21 | 2,810,968 | 57 | 147,622 | 327 | 452,757 | 33.04 |
| 28 | 7-31 | 2,784,800 | 26 | 353,754 | 315 | 1,018,014 | 33.10 |
| 29 | S2 | 2,750,537 | 44 | 139,305 | 327 | 451,925 | 33.12 |
| 30 | S6 | 2,815,784 | 62 | 154,024 | 316 | 452,672 | 33.2 |
| 31 | S7 | 2,792,276 | 47 | 152,069 | 327 | 452,164 | 33.26 |
| 32 | S20 | 2,851,372 | 71 | 147,725 | 327 | 452,740 | 33.06 |
| 33 | S21 | 2,777,880 | 58 | 154,018 | 327 | 450,958 | 33.24 |
| 34 | S27 | 2,784,910 | 57 | 147,449 | 317 | 452,387 | 33.13 |
| 35 | S29 | 2,750,537 | 44 | 168,164 | 327 | 451,925 | 33.03 |
| 36 | S31 | 2,814,780 | 59 | 153,773 | 317 | 452,533 | 33.06 |
| 37 | S33 | 2,785,010 | 59 | 149,331 | 317 | 452,813 | 32.99 |
| 38 | Y3 | 2,774,629 | 47 | 167,947 | 327 | 380,138 | 33.24 |
| 39 | Y6 | 2,727,236 | 45 | 153,764 | 327 | 452,778 | 33.36 |
| 40 | Y7 | 2,796,690 | 61 | 127,862 | 327 | 452,612 | 33.31 |
| 41 | Y12 | 2,804,953 | 56 | 168,179 | 317 | 452,789 | 33.25 |
| 42 | Y13 | 2,770,021 | 49 | 167,947 | 317 | 380,137 | 33.24 |
| 43 | S23 | 2,725,459 | 42 | 340,740 | 307 | 564,509 | 33.07 |
| 44 | S25 | 2,745,828 | 40 | 188,626 | 307 | 564,445 | 33.03 |
| 45 | Y1 | 2,838,762 | 65 | 105,783 | 327 | 315,558 | 33.29 |
| 46 | S4 | 2,798,875 | 84 | 81,392 | 315 | 253,079 | 32.97 |
| 47 | S30 | 2,784,351 | 25 | 509,043 | 315 | 1,018,070 | 33.09 |
| 48 | S8 | 2,730,496 | 48 | 127,596 | 315 | 298,417 | 33.35 |
| 49 | 5-2 | 2,767,532 | 45 | 168,019 | 327 | 452,789 | 33.20 |
| 50 | 5-6 | 2,743,415 | 50 | 149,331 | 306 | 452,751 | 33.33 |
| 51 | 5-12 | 2,813,533 | 59 | 149,247 | 327 | 452,706 | 33.25 |
| 52 | 5-13 | 2,807,363 | 61 | 149,238 | 317 | 452,737 | 33.22 |
| 53 | 5-16 | 2,758,953 | 50 | 149,254 | 327 | 452,788 | 33.39 |
| 54 | 5-17 | 2,759,092 | 50 | 149,254 | 327 | 452,788 | 33.34 |
| 55 | 5-19 | 2,764,546 | 49 | 128,024 | 327 | 492,688 | 33.35 |
| 56 | 5-22 | 2,798,601 | 55 | 170,039 | 327 | 452,789 | 33.28 |
| 57 | 5-23 | 2,783,975 | 70 | 132,808 | 304 | 354,250 | 33.23 |
| 58 | 5-25 | 2,757,753 | 43 | 168,178 | 327 | 452,788 | 33.14 |
| 59 | 5-27 | 2,757,272 | 45 | 168,178 | 317 | 452,788 | 33.12 |
| 60 | 5-29 | 2,775,806 | 53 | 147,541 | 327 | 452,736 | 33.25 |
| 61 | 5-30 | 2,794,837 | 55 | 149,343 | 317 | 450,051 | 33.27 |
| 62 | 5-31 | 2,790,164 | 50 | 167,947 | 317 | 380,139 | 33.17 |
| 63 | 5-39 | 2,768,768 | 49 | 149,343 | 327 | 452,738 | 33.32 |
| 64 | 5-9 | 2,754,459 | 30 | 305,172 | 325 | 420,941 | 33.18 |
| 65 | 5-15 | 2,754,317 | 28 | 305,172 | 325 | 461,684 | 33.29 |
| 66 | 5-14 | 2,764,051 | 33 | 637,371 | 308 | 1,048,534 | 33.24 |
| 67 | 7-13 | 2,824,874 | 66 | 94,627 | 372 | 199,169 | 33.13 |
| 68 | 1-7 | 2,752,897 | 43 | 168,645 | 327 | 449,999 | 33.34 |
| 69 | 1-25 | 2,770,898 | 51 | 149,343 | 327 | 452,712 | 33.25 |
| 70 | 2-6 | 2,761,328 | 75 | 153,774 | 322 | 449,807 | 33.23 |
| 71 | 2-32 | 2,810,928 | 65 | 149,347 | 327 | 452,705 | 33.32 |
| 72 | 2-39 | 2,787,732 | 43 | 169,700 | 317 | 452,735 | 33.23 |
| 73 | 3-16 | 2,805,830 | 65 | 127,673 | 327 | 450,009 | 33.31 |
| 74 | 1-6 | 2,861,969 | 57 | 185,388 | 315 | 402,430 | 33.18 |
| 75 | 1-19 | 2,862,044 | 60 | 151,244 | 315 | 365,087 | 33.09 |
| 76 | 2-12 | 2,862,396 | 59 | 169,777 | 315 | 402,362 | 32.95 |
| 77 | 3-5 | 2,865,925 | 56 | 233,294 | 315 | 587,400 | 32.93 |
| 78 | 4-3 | 2,861,905 | 61 | 184,610 | 315 | 402,542 | 33.07 |
| 79 | 4-10 | 2,855,094 | 55 | 151,244 | 315 | 464,278 | 33.13 |
| 80 | 4-9 | 2,802,593 | 45 | 338,484 | 307 | 627,608 | 33.40 |
| 81 | 1-22 | 2,813,910 | 56 | 149,343 | 327 | 452,616 | 33.09 |
| 82 | 2884 | 2,813,493 | 57 | 153,774 | 317 | 452,789 | 33.09 |
| 83 | 2885 | 2,767,830 | 49 | 149,253 | 317 | 452,762 | 33.03 |
| 84 | 3115 | 2,816,091 | 57 | 168,179 | 318 | 452,789 | 33.17 |
| 85 | 3116 | 2,813,474 | 56 | 153,774 | 317 | 452,768 | 33.11 |
| 86 | 3117 | 2,814,016 | 59 | 147,646 | 318 | 452,768 | 33.26 |
| 87 | 3118 | 2,817,062 | 57 | 155,172 | 317 | 452,768 | 33.13 |
| 88 | 2886 | 2,751,333 | 45 | 158,954 | 315 | 505,327 | 33.13 |
| 89 | 2887 | 2,801,640 | 45 | 274,868 | 315 | 341,403 | 32.99 |
| 90 | 2888 | 2,840,040 | 52 | 226,749 | 303 | 371,427 | 32.98 |
| 91 | 2-14 | 2,858,709 | 87 | 228,543 | 307 | 340,624 | 33.48 |
| 92 | 4-21 | 2,849,884 | 49 | 275,299 | 307 | 627,608 | 33.33 |
| 93 | 3114 | 2,788,927 | 25 | 418,519 | 315 | 652,953 | 33.22 |
