# Supplementary material for: Genome-based surveillance reveals cross-transmission of MRSA ST59 between humans and retail livestock products in Hanzhong, China
Source: Front Microbiol. 2024 Apr 29;15:1392134. doi: 10.3389/fmicb.2024.1392134 (PMC11089119; doi:10.3389/fmicb.2024.1392134)
Supplement: Supplementary file 4 [file Table_4.docx]

**Supplementary Table 4** Information of the online isolates used in phylogenetic analysis

|  | **Isolates** | **Geographic region** | **Origin** | **MLST** | ***spa* type** | **SCC*mec*** | **Genebank Accession** |
| --- | --- | --- | --- | --- | --- | --- | --- |
| 1 | 24-0 | Guangzhou | Retail meat | ST59 | t437 | IVa | WLAX00000000 |
| 2 | 24-1 | Guangzhou | Retail meat | ST59 | t437 | IVa | WLAY00000000 |
| 3 | 3025 | Xining | Retail meat | ST59 | t437 | IVa | WLAA00000000 |
| 4 | 522 | China | Retail meat | ST59 | t437 | Vb | WKZR00000000 |
| 5 | L18 | Guangzhou | human | ST59 | t437 | IVa | WLCE00000000 |
| 6 | M1 | Ningxia | human | ST59 | t441 | IVa | GCA_001412275.1 |
| 7 | P1 | Hongkong | human | ST59 | t441 | Vb | GCA_004168915.1 |
| 8 | SA40 | China (Taiwan) | Human | ST59 | t441 | IV | CP003604 |
| 9 | A69 | China | Swine | ST9 | t899 | XII | JJOP01000000 |
| 10 | AH027 | China | Human | ST9 | t899 | XII | GCA_003310835.1 |
| 11 | LYJ002 | China | Human | ST9 | t899 | XII | GCA_003693275.1 |
| 12 | M3 | China | Swine | ST9 | t899 | XII | MWRY00000000 |
| 13 | QD-CD9 | China | Swine | ST9 | t899 | XII | CP031838 |
| 14 | SAV1150 | Germany | poultry meat | ST9 | t899 | IV | QYAS01000000 |
| 15 | SAV1228 | Czech Republic | Poultry meat | ST9 | t899 | IV | QYAQ01000000 |
| 16 | TSAR03 | China (Taiwan) | Human | ST9 | t899 | XII | NADC00000000 |
| 17 | 08S00974 | Germany | Swine | ST398 | t011 | Vc | CP020019 |
| 18 | 10152 | Brazil | Human | ST398 | t034 | V | NZ_LZQL01000000 |
| 19 | Chi_10 | Austria | Chicken | ST398 | t011 | Vc | JIYV00000000 |
| 20 | FY22 | China | Human | ST398 | t034 | V | NXFU00000000 |
| 21 | GD5 | China | Human | ST398 | t034 | Vb | CP019592 |
| 22 | GDC6P096P | China | Swine | ST398 | t034 | Vc | CP065194 |
| 23 | PTDrAP2 | Australia | Swine | ST398 | t034 | Vc | CP029172 |
| 24 | SAV1146 | Germany | Poultry meat | ST398 | t899 | IVa | QYAU00000000 |
